# Direct Comparison of Immunogenicity Induced by 10- or 13-Valent Pneumococcal Conjugate Vaccine around the 11-Month Booster in Dutch Infants

**DOI:** 10.1371/journal.pone.0144739

**Published:** 2015-12-10

**Authors:** Alienke J. Wijmenga-Monsuur, Els van Westen, Mirjam J. Knol, Riet M. C. Jongerius, Marta Zancolli, David Goldblatt, Pieter G. M. van Gageldonk, Irina Tcherniaeva, Guy A. M. Berbers, Nynke Y. Rots

**Affiliations:** 1 Centre for Infectious Disease Control, National institute for Public Health and the Environment, Bilthoven, The Netherlands; 2 University College London, Institute of Child Health, London, United Kingdom; The George Washington University School of Medicine and Health Sciences, UNITED STATES

## Abstract

**Background & Aims:**

Since 2009/10, a 10- and a 13-valent pneumococcal conjugate vaccine (PCV) are available, but only the 10-valent vaccine is now being used for the children in the Netherlands. As the vaccines differ in number of serotypes, antigen concentration, and carrier proteins this study was designed to directly compare quantity and quality of the antibody responses induced by PCV10 and PCV13 before and after the 11-month booster.

**Methods:**

Dutch infants (n = 132) were immunized with either PCV10 or PCV13 and DTaP-IPV-Hib-HepB at the age of 2, 3, 4 and 11 months. Blood samples were collected pre-booster and post-booster at one week and one month post-booster for quantitative and qualitative immunogenicity against 13 pneumococcal serotypes, as well as quantitative immunogenicity against diphtheria, tetanus, pertussis and *Haemophilus influenzae* type b. We compared immunogenicity induced by PCV13 and PCV10 for their ten shared serotypes.

**Results:**

One month post-booster, pneumococcal serotype-specific IgG geometric mean concentrations (GMCs) for the PCV13 group were higher compared with the PCV10 group for six serotypes, although avidity was lower. Serotype 19F showed the most distinct difference in IgG and, in contrast to other serotypes, its avidity was higher in the PCV13 group. One week post-booster, opsonophagocytosis for serotype 19F did not differ significantly between the PCV10- and the PCV13 group.

**Conclusion:**

Both PCV10 and PCV13 were immunogenic and induced a booster response. Compared to the PCV10 group, the PCV13 group showed higher levels for serotype 19F GMCs and avidity, pre- as well as post-booster, although opsonophagocytosis did not differ significantly between groups. In our study, avidity is not correlated to opsonophagocytotic activity (OPA) and correlations between IgG and OPA differ per serotype. Therefore, besides assays to determine IgG GMCs, assays to detect opsonophagocytotic activity, i.e., the actual killing of the pneumococcus, are important for PCV evaluation. How differences between the two vaccines relate to long-term protection requires further investigation.

**Trial Registration:**

www.trialregister.nl NTR3069

## Introduction

*Streptococcus pneumoniae (SP)* is an important cause of morbidity and mortality worldwide, with the highest disease incidence among children under 2 years of age. Carriage of *SP* is often asymptomatic but can result in non-invasive mucosal infections or invasive pneumococcal disease (IPD). More than 90 SP serotypes of have been identified, of which about 20 serotypes tend to cause IPD [1–3]. The 2006 introduction of a 7-valent pneumococcal conjugate vaccine (PCV7) for infants in the Netherlands dramatically decrease SP carriage and IPD for its seven serotypes [4–10], in line with other countries that implemented PCV7 [11, 12]. However, carriage and IPD related to serotypes not covered by PCV7 have increased since its introduction of PCV7 [4–7, 9, 10]. In 2009, two new pneumococcal conjugate vaccines were licensed that provide protection against 10 (PCV10) or 13 (PCV13) serotypes. They share serotypes 1, 4, 5, 6B, 7F, 9V, 14, 18C, 19F and 23F, and PCV13 in addition includes 3, 6A and 19A. In the Netherlands, all children born since March 2011 have received PCV10 instead of PCV7. Carriage of the three additional serotypes of PCV10 (1, 5 and 7F) was already low before its introduction, and scarcely changed in the first 1.5 year afterward [9]. However, overall IPD incidence decreased in the youngest age group (<2 years), and some decrease has been seen in adults >50 years of age [10]. Even if PCV10 should prove superior to PCV7, the question remains whether PCV13 might offer additional improvement. Besides differing in the number of serotypes, PCV10 and PCV13 differ in the carrier protein and the amount of antigen per serotype, factors that might influence the induction of B-cell responses and consequently antibody responses [13–19]. Of the PCV10 serotypes, 8 are conjugated to protein D of non-typeable *H*. *influenzae*, while 18C is conjugated to tetanus toxoid and 19F is conjugated to diphtheria toxoid. For PCV7 and PCV13, the carrier protein is CRM197, a non-toxic variant of diphtheria toxin.

Although PCV10 and PCV13 have both been compared with PCV7 as to induction of immunogenicity [20–26], to our knowledge no direct comparison between PCV10 and PCV13 for all serotypes has been published. In this study we evaluate not only the quantity of the antibodies (the serotype-specific total IgG antibody concentrations), but also the quality or functional activity of the antibodies. This was evaluated through analysis of avidity, i.e., the strength of antibody-antigen binding interactions, and opsonophagocytosis, i.e., the ability to opsonize bacteria to facilitate phagocytosis.

The current study compared, head-to-head, PCV10 and PCV13 administered in the primary series (at 2, 3 and 4 months) and as a booster dose at 11 months of age. Here we report the immunogenicity data, including pneumococcal serotype-specific IgG antibody concentrations and avidity in serum samples, and opsonophagocytosis responses in plasma samples, obtained pre-booster and post-booster at one week and one month. In addition to pneumococcal data, serum IgG concentrations against diphtheria, tetanus, pertussis and *H*. *influenzae* type b were compared. Our primary objective was to compare immunogenicity at one month post-booster induced by immunizations with PCV10 or PCV13. Secondary objectives were: 1) to compare immunogenicity pre-booster and one week post-booster induced by immunizations with PCV10 or PCV13, 2) to investigate pre-booster and post-booster the possible influence of PCV10 or PCV13 immunizations on the immune responses induced by diphtheria, tetanus, pertussis and *H*. *influenzae* type b.

## Material and Methods

### Study design

**Fig 1:**
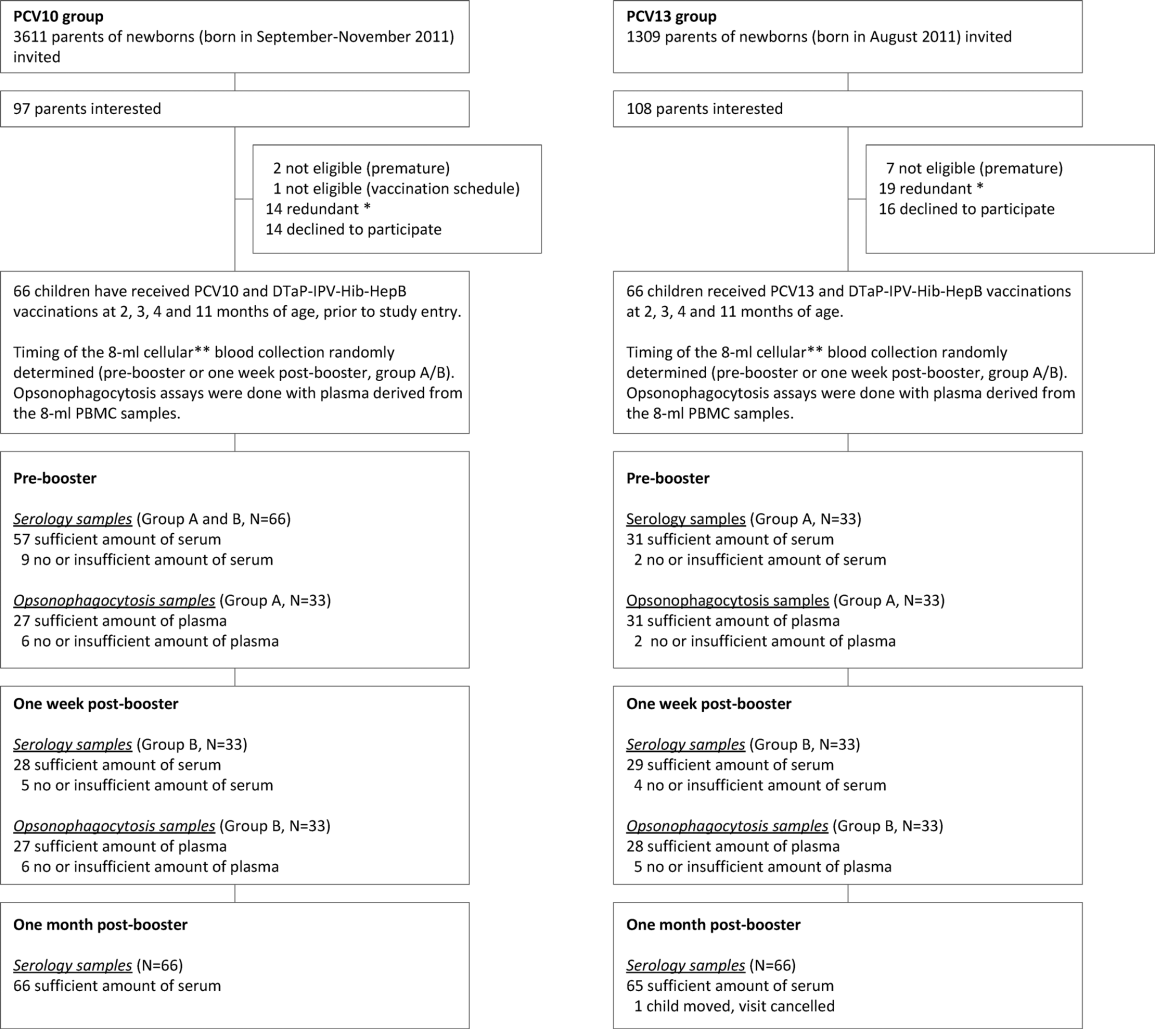
Enrollment and Study Participation. PCV10 indicates 10-valent pneumococcal conjugate vaccine, Synflorix; PCV13 indicates 13-valent pneumococcal conjugate vaccine, Prevenar-13. * When the enrolment target was reached, further children were considered redundant, and their parents’ informed consent procedures were cancelled. ** Plasma cell and memory B-cell analysis published by van Westen et al.[27].

A single-centre, parallel-group intervention study with two groups (PCV10 recipients and PCV13 recipients) was conducted in the Netherlands among infants eligible for the routine National Immunization Program (NIP): vaccinations at 2, 3, 4 and 11 months of age, or the 3+1 (primary plus booster) schedule (S1 Text). Through their parents, infants born in August 2011 were invited for the PCV13 group, and infants born in September-November 2011 were invited for the PCV10 group (Fig 1). Exclusion criteria were a serious and/or immunological disease that could interfere with the results of the study; previous administration of plasma products; presence of bleeding disorders, and prematurity (gestational age <37 weeks). No financial compensation for participants was provided. Infants in the PCV10 group received Synflorix (GlaxoSmithKline Biologicals s.a., Rixensart, Belgium) during regular well-baby clinic visits according to the NIP schedule. Infants in the PCV13 group received Prevenar-13 (Pfizer Limited, Sandwich, Hampshire, UK) during home visits by the study team according to the NIP schedule. Both groups received concomitantly DTaP-IPV-Hib-HepB (Infanrix hexa, GlaxoSmithKline Biologicals s.a., Rixensart, Belgium).

For B-cell and immunogenicity assays, an 8-ml blood sample was collected by venipuncture and randomly allocated “pre-booster” for half of the children (group A) and “one week post-booster” for the other half (group B). From children of the PCV10 group, who gave the 8-ml post-booster sample, an additional pre-booster sample (300 µl) was collected by heel stick. In all subjects, to collect the one month post-booster blood (300 µl), which was the primary endpoint, the heel stick was used because it was easier for the children. This was an open-label study for parents and study staff, but immunogenicity analysis was performed blinded. An acknowledged national ethics committee approved the study (METC Noord Holland, NTR3069). All study-related activities were performed in accordance with European guidelines for Good Clinical Practice, which includes provisions of the Declaration of Helsinki, with written informed consent from the child’s parents or the guardian.

In the current article, we publish the quantitative IgG data for all 132 subjects, of all three time-points (pre-booster, one week post-booster, and one month post-booster) and all 13 serotypes. In addition, we publish the qualitative data, i.e., avidity and opsonophagocytosis of pneumococcal serotypes and IgG data of diphtheria, tetanus, pertussis and *H*. *influenzae* type b. Results on the B-cell assays were previously published, open access, by van Westen et al. [27] who also included IgG data for a subset of the children for 6 of the 13 serotypes, pre-booster and one week post-booster.

### Humoral analyses

After blood collection, sera for serological analyses and plasma for opsonophagocytosis assays were separated and stored at -80°C. Pneumococcal serotype-specific IgG antibody concentrations against the 13 pneumococcal serotypes were measured using a fluorescent bead-based multiplex immunoassay (MIA), as described previously [28]. Avidity for all 13 serotypes was determined with a MIA using 0.5 M ammonium isothiocyanate (NH_4_SCN) compared with phosphate buffered saline (PBS) [29]. Opsonophagocytosis activity (OPA) was determined for all 13 serotypes by multiplex opsonophagocytosis assay performed at UCL Institute of Child Health (London, UK) [30]. Antibody concentrations against DTaP-Hib antigens were determined by MIA, as described previously [31–33].

### Statistical analyses

In this type of study, a 2- to 2.5-fold difference is considered to represent a true difference in immunogenicity [20, 34]. A minimum of 47 children per group were needed in order to provide 80% power to detect a 2-fold difference in IgG geometric mean concentrations (GMCs) between the two groups, with an assumed variance of the log-transformed concentration of 0.27 (based on serotype 6B, the serotype with the highest variance). The following formula was used n = 2k^2^σ^2^ / δ^2^. Since 66 children per group had already participated in the study for the B-cell assays [27], a 1.8-fold difference could be detected at one month post-booster the primary endpoint. For the other time-points, collected blood samples were fewer but sufficient to detect a 2- to 2.5-fold difference. Baseline characteristics including sex, age at vaccination and blood sampling, day-care attendance at 12 months of age, birth-weight, gestational age, presence of siblings < 5 year of age, duration of breast-feeding, and passive smoking were compared between the two groups, using a t-test for continuous variables and a chi-square test for categorical variables.

For each group, pneumococcal serotype-specific IgG GMCs, mean avidity indices (MAI), and OPA geometric mean titers (GMTs) with 95% confidence intervals (CI) were calculated. Percentages of vaccine recipients reaching an IgG threshold of 0.35 μg/ml for all time-points and 1.0 μg/ml post-booster were calculated. IgG GMCs were calculated diphtheria, tetanus, pertussis and *H*. *influenzae* type b antigens, and their internationally accepted thresholds for protection were used for seroprotection rate analysis. Differences in IgG GMCs, MIA, and OPA GMTs between groups were analyzed with a t-test, and differences in the percentage of subjects reaching a threshold were tested with a chi-square test or, when appropriate, a Fisher’s Exact test. Spearman correlations between IgG and OPA or avidity and OPA were calculated. For analyses between post-booster time-points (one week versus one month), a paired t-test was used.

Since the age at vaccination and blood sampling differed slightly between the groups, we performed secondary analyses in which we adjusted for age in days at vaccination and blood sampling using regression analysis.

All data were analyzed using SPSS 19. A p value of <0.05 was considered statistically significant.

## Results

### Study population

In total, 132 infants were enrolled in the study, both groups (PCV10 recipients and PCV13 recipients) consisting of 66 infants (Fig 1). Baseline characteristics of the two groups are summarized in Table 1. Subjects of the PCV10 group, immunized during regular well-baby clinic visits, were slightly older at vaccination and blood sampling than children of the PCV13 group, who were vaccinated at home by the study team. This difference reflects the normal delays that occur when families plan clinic visits. Compared to the standard analyses described below, regression analyses for all time-points, with adjustment for age at vaccination and blood sampling (S1–S3 Tables), showed no change in the pattern of differences between the PCV13 group and the PCV10 group, although some differences were no longer statistically significant.

**Table 1:** Characteristics of the participants.

	PCV13 (n = 66)	PCV10 (n = 66)	p-value
Male sex, %	62.1	50.0	0.161
Month of birth, median (min-max)	Sept (Sept-Sept)	Nov (Oct-Dec)	-
Day-care attendance^a^, %	81.5^b^	78.8	0.693
Half-days at day-care^a^, mean (sd)	4.5 (1.6)	4.7 (1.7)	0.568
Birth-weight in grams, mean (sd)	3578 (458)	3636 (471)	0.477
Gestational age in weeks, mean (sd)	39.6 (1.3)	39.8 (1.3)	0.579
Presence of siblings < 5 years, %	40.9	50.0	0.294
Breast-feeding, %	81.8	81.8	1.000
Duration of breast-feeding in months, mean (sd)	5.6 (3.0)	6.3 (3.7)	0.309
Passive smoking, %	0.0	1.5	0.315
Age at 2-month vaccination, mean (sd)	1.9 (0.12)	2.0 (0.17)	<0.001

a At 12 months of age

### One month post-booster immunogenicity for the ten shared pneumococcal serotypes

**Fig 2:**
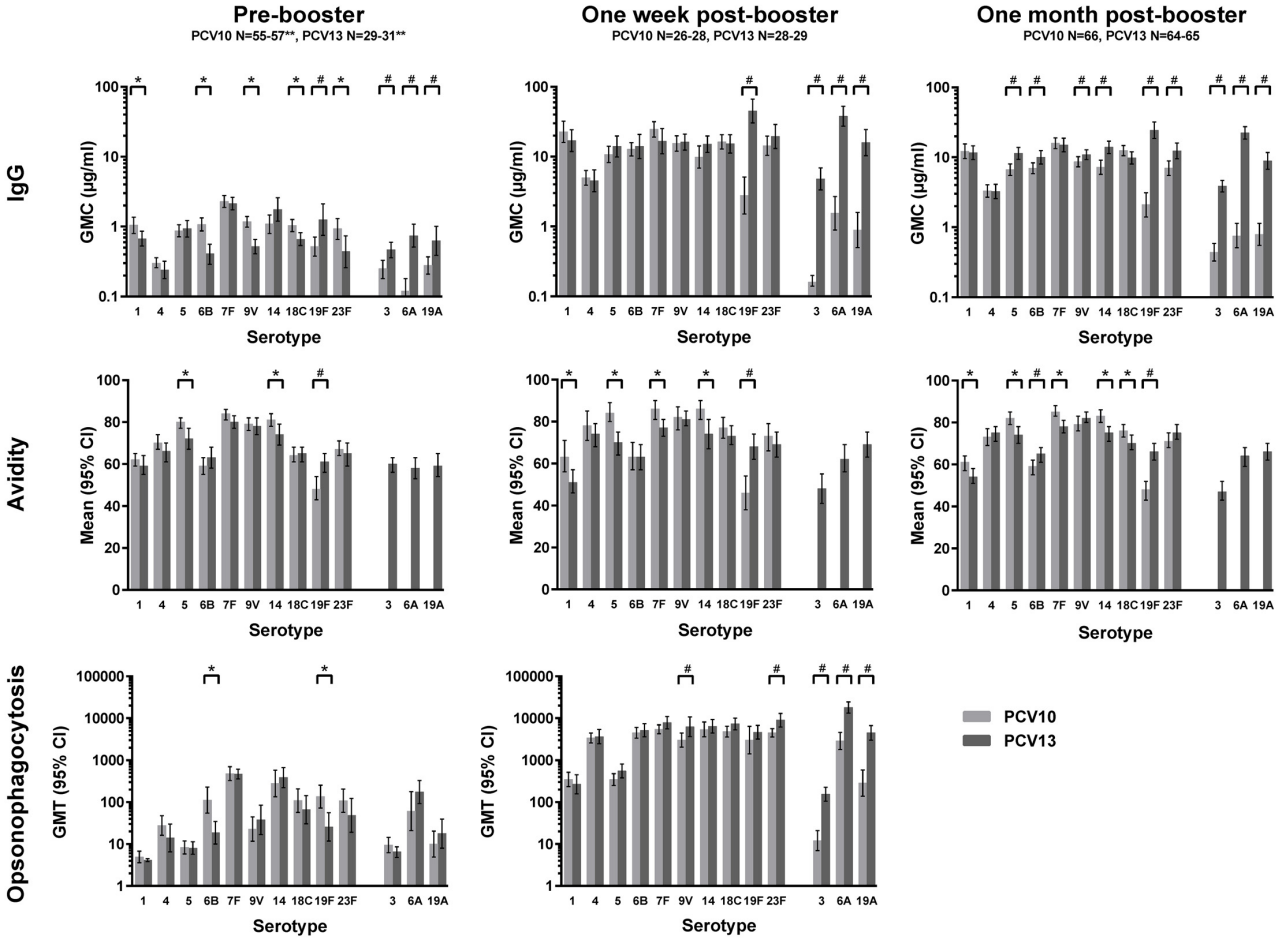
Pneumococcal serotype-specific measurements. IgG geometric mean concentrations (GMCs) pre-booster, one week post-booster and one month post-booster; Mean avidity indices of IgG antibodies pre-booster, one week post-booster, and one month post-booster; opsonophagocytosis geometric mean titers (GMTs) pre-booster and one week post-booster. *indicates significant differences (<0.05), higher in the PCV10 group than the PCV13 group ^#^indicates significant differences (<0.05), higher in the PCV13 group than the PCV10 group **sample size for opsonophagocytosis assays: PCV10 group N = 22–27, PCV13 group N = 25–31

One month post-booster, IgG GMCs were higher for serotypes 5, 6B, 9V, 14, 19F and 23F in the PCV13 group compared with the PCV10 group (Fig 2 and S1 Table). Avidity indices of IgG antibodies induced by PCV13 post-booster were higher for serotype 6B and 19F, but lower for 5 serotypes compared with the PCV10 group (Fig 2 and S2 Table). Nearly all participants reached the 0.35 μg/ml seroprotection threshold for all serotypes except for 19F in the PCV10 group (86%) (Table 2). The percentage of children with antibody concentrations >1.0 μg/ml was >90% for most serotypes, except in the PCV13 group for serotype 4 (89%) and in the PCV10 group for serotype 19F (73%).

**Table 2:** Seroprotection rate of the antibody concentrations against 13 pneumococcal serotypes for the PCV10 group and the PCV13 group with p-values for differences between the groups.

	Pre-booster			One month post-booster					
	PCV13 (N = 31)	PCV10 (N = 57)		PCV13 (N = 65)		PCV10 (N = 66)			
Serotype	>0.35	>0.35	p-value >0.35	>0.35	>1.0	>0.35	>1.0	p-value* >0.35	p-value* >1.0
1	77	88	0.207	100	100	100	97	-	0.496
4	29	39	0.370	97	89	100	92	0.244	0.561
5	94	90	0.707*	100	99	100	100	-	0.496
6B	68	93	0.002	99	99	100	100	0.496	0.496
7F	100	97	0.538*	99	99	100	100	0.496	-
9V	77	97	0.008*	100	100	100	100	-	-
14	94	90	0.707*	100	100	100	96	-	0.244
18C	84	95	0.124*	100	99	100	100	-	0.496
19F	87	65	0.026	100	100	86	73	0.003	<0.001
23F	55	83	0.005	99	99	100	94	0.496	0.365
3	61	30	0.004	100	95	55	23	<0.001	<0.001
6A	81	19	<0.001	100	100	76	44	<0.001	<0.001
19A	71	42	0.010	100	92	76	32	<0.001	<0.001

* Fisher Exact test

### Pre-booster immunogenicity for the ten shared pneumococcal serotypes

Compared with the PCV10 group, pre-booster IgG GMCs were lower in the PCV13 group for 5 of the 10 shared serotypes but higher for serotype 19F (Fig 2 and S1 Table). Avidity indices of the PCV13 group were lower for serotypes 5 and 14 but higher for 19F, compared with the PCV10 group (Fig 2). Functional OPA GMTs in the PCV13 group were lower for serotype 6B and 19F (Fig 2 and S3 Table). In contrast, the IgG GMC and avidity index for serotype 19F were higher for PCV13-vaccinated children compared with PCV10-vaccinated children. The percentage of children with antibody concentrations >0.35 μg/ml was above 75% for most serotypes (Table 2).

### One week post-booster immunogenicity for the ten shared pneumococcal serotypes

Surprisingly, all one week post-booster IgG GMCs were significantly higher (p<0.002) than one month post-booster IgG GMCs (p values not shown), except for serotype 19F in the PCV10 group. Differences in IgG GMC and avidity data between the PCV13 and PCV10 groups were similar at one week and one month. However, in the one week post-booster groups, which included only half of the children of the PCV10 and PCV13 groups, the differences were in most cases not significant (Fig 2 and S1 and S2 Tables). OPA GMTs were significantly higher in the PCV13 group for serotypes 9V and 23F (Fig 2 and S3 Table). Spearman correlations were serotype-specific and found only found between IgG GMC and OPA; no correlation between avidity and OPA or IgG was found except for PCV10 serotype 1 (S5 Table and S1 Fig).

### PCV13-specific serotypes 3, 6A and 19A

PCV13 induced IgG GMCs for serotype 3, 6A and 19A with pre-booster seroprotection rates ranging from 61% to 81% whereas the rates one month post-booster were 100% for antibody concentrations >0.35 μg/ml and 92–100% for antibody concentrations >1.0 μg/ml (Table 2 and S1 Table). PCV13 avidity indices these serotypes were comparable to other serotypes (Fig 2 and S2 Table), for all three time-points. In the PCV10-group IgG GMCs were slightly increased at one week and one month post-booster, compared with pre-booster, for serotypes 3, 6A and 19A (Fig 2, S1 Table), but avidity was not measured. One week post-booster in the PCV10 group, OPA GMTs had increased for serotypes 6A and 19A but were much lower than for the PCV13 group (S3 Table).

### One month post-booster immune response of DTaP-Hib

Diphtheria- and pertussis-specific IgG GMCs and seroprotection rates induced by the concomitantly given DTaP-Hib vaccine did not differ between the groups (Table 3 and S4 Table). In both groups, all vaccine components showed a good booster response. However, Tetanus-specific IgG GMCs were significantly lower in the PCV13 group than the PCV10 group, whereas seroprotection rates were similar. Hib IgG GMCs were only significantly lower in the PCV13 group compared with the PCV10 group in the age-adjusted analyses.

**Table 3:** Seroprotection rate (%) for diphtheria, tetanus, pertussis, and Hib antigens for the PCV10 group and the PCV13 group with p-values for differences between the groups.

	Pre-booster			One month post-booster		
Antigen	PCV13 (N = 31)	PCV10 (N = 56)	p-value	PCV13 (N = 65)	PCV10 (N = 66)	p-value
Diphtheria > = 0.10 IU/ml	68	63	0.625	100	100	1.000
Pertussis PT > = 20 EU/ml	39	39	0.958	99	100	0.496*
Tetanus > = 0.10 IU/ml	90	100	0.042*	100	100	1.000
Hib > = 0.15 mcg/ml	21**	68	<0.001*	97	100***	0.496*

*Fisher Exact test

**N = 29 for PCV13

***N = 65 for PCV10

## Discussion

The current study compared immunogenicity induced by PCV13 and PCV10 given at 2, 3, 4 and 11 months of age in a Dutch cohort of 132 infants. In addition to the quantitative antibody levels (IgG), also the quality of these IgG antibodies was examined by measuring avidity and functional opsonophagocytic activity (OPA) for the pneumococcal vaccine serotypes before and after the booster vaccination.

### Post-booster data

Post-booster, the IgG GMC levels for six shared serotypes were higher for the PCV13 group than for the PCV10 group. The PCV13 group also had higher functional OPA responses for two serotypes but higher avidity levels only for one serotype. The age at vaccination and blood sampling differed slightly between the groups, and the vaccination schedule varied more in the PCV10 group. However, the age differences between the groups did not appear to affect immune responses, as the age-adjusted analysis showed similar post-booster data for both products. Likewise, the results of the study of Spijkerman et al. showed that four different primary PCV13 vaccination schedules resulted in similar one month post-booster immunogenicity outcomes [29]. Differences found between our two groups could therefore be due to actual differences between the two PCVs, such as serotype concentrations, conjugates, and additives.

The most pronounced difference in immunological response between the two vaccines was found for serotype 19F, which showed a significantly higher IgG GMC and avidity index in the PCV13 group compared with PCV10 group. In our study, however, the serotype 19F-specific IgG GMC (2.09 μg/ml) induced by PCV10 one month post-booster was much lower than found in previous studies (4.23 to 9.72 μg/ml) [20, 21, 35–39]. Also, the related seroprotection rate (>0.35 μg/ml), which was 86% for serotype 19F for our PCV10 group, is lower than the 97–100% reported in other studies [20, 21, 35–39]. Seroprotection rates are based on internationally accepted ELISA IgG GMCs cut-off levels of >0.35 μg/ml for all serotypes [40]. Recently, Andrews et al. proposed serotype-specific ELISA thresholds for protection based on effectiveness data for PCV13 [41]. For serotype 19F, they determined a threshold of 1.17 μg/ml, which would translate to a serotype 19F-specific seroprotection rate of 70% for our PCV10 group and 100% for the PCV13 group one month post-booster. Moreover, our seroprotection rate was calculated using IgG GMCs determined in a MIA, which in general results in slightly higher levels than the WHO ELISA [28]. Serotype 19F IgG GMCs for our PCV13 group, analyzed in same assay run as for PCV10, indeed were relatively high (24.37 μg/ml), especially compared with data generated using the WHO ELISA. This finding indicates that the already low values for the PCV10 group might have been even lower if the WHO ELISA had been used. In spite of low serotype 19F IgG GMCs and avidity for the PCV10 group, OPA responses and OPA seroprotection rates were comparable to those in the PCV13 group, They were 88.9% and 100% for the PCV10 group and the PCV13 group, respectively, when using the 19F-specific seroprotection cut-off level of 430 [41]. This suggests a low correlation between IgG and OPA, which was indeed only 0.39 in the PCV10 group and 0.49 in the PCV13 group. The relatively high serotype 19F OPA response in the PCV10 group, despite the low IgG results could be explained by a higher IgM antibody response, since IgM is be effective at opsonizing bacteria [42, 43]. Moreover, the timing of the OPA samples in this study should be considered as we measured at one week post-booster, which could reflect the recall of the memory response, whereas if we would have measured at one month post-booster the time-point normally used for IgG antibodies, our measurements could have reflected a mixture of memory and newly generated antibodies.

Protection against disease by PCV10 seems adequate, SP carriage has remained low (1%) since its 2009 introduction in the Netherlands and no PCV10 serotype 19F vaccine failures have been observed [9, 44] despite low serotype 19F IgG GMCs and avidity.

Studies in Finland and Brazil have likewise found PCV10 to be 84–100% effective against IPD [45, 46].

For PCV13 serotype 19F, the current study showed an IgG GMC of 24.37 μg/ml, which is higher than in previous studies of both PCV7 (2.4–5.6 μg/ml)[20, 22, 23, 38, 47] and PCV13studies (6.6–11.06 μg/ml)[22, 23, 47] and could be related to the use of MIA instead of ELISA [28]. However, Grant et al. and Dagan et al. [22, 23], using ELISA, also reported higher 19F-specific responses for PCV13 than seen in previous PCV7 studies. Cross-reaction by serotype 19A could play a role [22, 48–50]. Overall, the reported higher serotype 19F-specific IgG GMCs, avidity, and OPA induced in our PCV13 group could result in better protection against pneumococcal disease than induced by PCV7, for which six vaccine failures in 5 years occurred in the Dutch population [44].

Van Westen et al. published the plasma and memory B-cells results of described trial for 6 serotypes, showing no clear correlation between IgG and plasma or memory B-cells. In our paper, we show a weak correlation between IgG GMCs and OPA and no correlation between avidity and OPA. Together with the fact that OPA measures actual killing of the bacteria and avidity index is a measure of antigen-antibody binding strength implies that for comparison of differences in vaccine-induced immunogenicity, IgG GMCs combined with OPA analysis should be performed.

### Pre-booster data

Pre-booster seroprotection rates for pneumococcal vaccine serotypes remained high for both our groups, indicating that protection of children is adequate during the whole period between the primary series and the booster dose. Whether the 5 serotypes with lower IgG GMCs pre-booster in the PCV13 group, compared with PCV10, reflect a lower initial response after the primary series or a steeper decline cannot be concluded from data collected in this trial.

Interestingly, Spijkerman et al. showed that pre-booster antibody levels at 11 months of age were higher when the primary vaccinations started at an older age (three versus two months) or had longer intervals between doses (two versus one month) [29]. Children in our PCV13 group followed the NIP schedule for the primary vaccinations more strictly than the children in the PCV10 group, who in general had somewhat longer intervals between the doses. This might explain the differences between the groups in pre-booster GMCs at 11 months. However, the differences in the intervals are relatively small (at most 0.4 months at the third vaccination), and a more plausible explanation for the results is therefore the difference in vaccine composition between PCV13 and PCV10. Despite somewhat lower IgG GMCs for the PCV13 group, compared with the PCV10 group, PCV13 is still expected to be effective in the period between primary and booster vaccination. Its effectiveness has been shown in countries where it has been implemented, and even with herd protection induced by PCV7, a further decline of IPD caused by PCV7 serotypes is seen after introduction of PCV13 [51–53]. Also in other studies, comparable seroprotection rates for PCV13 at one month after the primary series have been shown [26, 29, 41].

### Added value of serotype 3, 6A and 19A

Although serotypes 3, 6A and 19A are not present in PCV10, IgG GMCs against these serotypes could be detected in the PCV10 group, pre- as well as post-booster dose. For serotype 6A and 19A, a small but significant increase in IgG GMCs was found after the booster dose along with functional post-booster OPA (S1 Table). Both findings could be a result of carriage of 6A and 19A serotypes by 9–15% of the Dutch children at 11 months [4, 5, 9] or cross-reactivity between serotype 6B and 6A and between 19F and 19A antibodies [22, 48–50]. However, post-PCV10-booster, IgG GMCs for these serotypes were 10-30-fold lower than the IgG GMCs reached in PCV13-vaccinated children, and only 9 of 27 PCV10-vaccinated children showed serotype 19A-specific OPA titers comparable to the OPA titers measured in PCV13-vaccinated children, all others being lower. One week post-booster, 89% of the children of the PCV10 group and 100% of the PCV13 group reached the serotype 19A-specific OPA seroprotection threshold of 48 calculated by Andrews et al. as protective against serotype 19A [41]. In the study published by van Westen et al., the PCV10 group had no post-booster increase in plasma and memory B-cells for serotype 6A and 19A, possibly because the cross-reactive vaccine serotypes did not generate an optimal memory response for the non-vaccine serotypes [27]. Whether PCV10 induces protection against serotype 19A disease remains to be seen.

### Influence on other NIP components

Serum IgG GMCs against the concomitantly given tetanus and *H*. *influenzae* type b (Hib) vaccine components were significantly lower in the PCV13 group compared with the PCV10 group. Enhancement of the antibody responses to Hib, diphtheria and tetanus vaccines by PCV10 vaccination have been reported [54] and could be related to the conjugate proteins used for this vaccine. The clinical relevance of these differences is unknown.

### Strength and limitations of this study

The strength of our study is the head-to-head comparison within one trial of immunogenicity induced by PCV10 and PCV13, with all samples analyzed in the same lab using the same methods. An additional strength is that post-booster responses were evaluated at two short-term time-points (one week and one month after the booster), and multiple immunogenicity parameters, including functional activity, were evaluated (IgG GMC, avidity and opsonophagocytosis), generating a more complete immunological profile. We could therefore also determine if IgG or avidity is a better predictor of vaccine effectiveness. A study limitation is the small difference in age at vaccination and blood sampling between the two groups, due to different vaccination practices (well-baby clinic visits for the PCV10 group and home visits for the PCV13 group); however, the age-adjusted analyses showed no difference in immunogenicity.

## Conclusion

Both PCV10 and PCV13 are immunogenic and able to induce a good booster response. In the PCV13 group compared to the PCV10 group, six shared serotypes showed higher IgG GMCs, and two higher avidity at one month post-booster, whereas pre-booster, five serotypes showed lower IgG GMCs. Pneumococcal serotype 19F-specific immune responses induced by PCV13 were higher than PCV10-induced responses, pre- as well as post-booster. Beyond the quantitative serum IgG GMC measurements, assessment of the qualitative activity of generated antibodies by avidity and OPA analysis showed the added value, particularly for serotype 19F. In general, IgG GMCs correlated better with opsonophagocytosis than avidity with opsonophagocytosis. Further investigation is required to determine the clinical relevance of the differences between PCV10- and PCV13-induced responses and their impact on SP carriage and long-term protection.

## Supporting Information

S1 Text Approved protocol. (PDF)

S2 Text Trend statement checklist. (PDF)

S1 Table Geometric mean concentrations (GMC) with 95% CI of the antibody concentrations against 13 pneumococcal serotypes for the PCV10 group and the PCV13 group. (PDF)

S2 Table Mean avidity index with 95% CI for 13 pneumococcal serotypes for the PCV10 group and the PCV13 group. (PDF)

S3 Table Geometric mean titers (GMT) with 95% CI of opsonophagocytosis for 13 pneumococcal serotypes for the PCV10 group and the PCV13 group. (PDF)

S4 Table Geometric mean concentrations (GMC) with 95% CI of the antibody concentrations against diphtheria, tetanus, pertussis and Hib antigens for the PCV10 group and the PCV13 group. (PDF)

S5 Table Spearman correlations one week post-booster between IgG, OPA titer and avidity for the PCV10 group and the PCV13 group. (PDF)

S1 Fig Spearman correlations one week post-booster between IgG, OPA titer and avidity for the PCV10 group and the PCV13 group. (PDF)

## References

[1] Oosterhuis-Kafeja F, Beutels P, Van Damme P. Immunogenicity, efficacy, safety and effectiveness of pneumococcal conjugate vaccines (1998–2006). Vaccine. 2007;25(12):2194–212. 10.1016/j.vaccine.2006.11.032 . 17267077

[2] Johnson HL, Deloria-Knoll M, Levine OS, Stoszek SK, Freimanis Hance L, Reithinger R, et al Systematic evaluation of serotypes causing invasive pneumococcal disease among children under five: the pneumococcal global serotype project. PLoS medicine. 2010;7(10). 10.1371/journal.pmed.1000348 20957191 PMC2950132

[3] van der Linden M, Reinert RR, Kern WV, Imohl M. Epidemiology of serotype 19A isolates from invasive pneumococcal disease in German children. BMC infectious diseases. 2013;13:70 10.1186/1471-2334-13-70 23384407 PMC3570384

[4] Spijkerman J, Prevaes SM, van Gils EJ, Veenhoven RH, Bruin JP, Bogaert D, et al Long-term effects of pneumococcal conjugate vaccine on nasopharyngeal carriage of S. pneumoniae, S. aureus, H. influenzae and M. catarrhalis. PloS one. 2012;7(6):e39730 10.1371/journal.pone.0039730 22761879 PMC3382588

[5] Spijkerman J, van Gils EJ, Veenhoven RH, Hak E, Yzerman EP, van der Ende A, et al Carriage of Streptococcus pneumoniae 3 years after start of vaccination program, the Netherlands. Emerging infectious diseases. 2011;17(4):584–91. 10.3201/eid1704.101115 21470445 PMC3377405

[6] van Deursen AM, van Mens SP, Sanders EA, Vlaminckx BJ, de Melker HE, Schouls LM, et al Invasive pneumococcal disease and 7-valent pneumococcal conjugate vaccine, the Netherlands. Emerging infectious diseases. 2012;18(11):1729–37. 10.3201/eid1811.120329 23092683 PMC3559145

[7] Rodenburg GD, de Greeff SC, Jansen AG, de Melker HE, Schouls LM, Hak E, et al Effects of pneumococcal conjugate vaccine 2 years after its introduction, the Netherlands. Emerging infectious diseases. 2010;16(5):816–23. 10.3201/eid1605.091223 20409372 PMC2953990

[8] Myint TT, Madhava H, Balmer P, Christopoulou D, Attal S, Menegas D, et al The impact of 7-valent pneumococcal conjugate vaccine on invasive pneumococcal disease: a literature review. Advances in therapy. 2013;30(2):127–51. 10.1007/s12325-013-0007-6 . 23397399

[9] Bosch AATM, Bruin JP, Wijmenga-Monsuur AJ, Trzcinski K, Bogaert D, Rots NY, et al Nasopharyngeal carriage of Streptococcus pneumoniae and other bacteria in the 7th year after implementation of the pneumococcal conjugate vaccine. Emerging infectious diseases. Submitted. 10.1016/j.vaccine.2015.11.060 26667610

[10] Knol MJ, Wagenvoort GHJ, E.A.M. S, Elberse K, Vlaminckx BJ, Melker de HE, et al Incidence of invasive pneumococcal disease in the Netherlands in the first three years after introduction of 10-valent pneumococcal conjugate vaccination. Emergin Infectious Diseases. Submitted. 10.3201/eid2111.140780 PMC4622227 26488415

[11] van Hoek AJ, Sheppard CL, Andrews NJ, Waight PA, Slack MP, Harrison TG, et al Pneumococcal carriage in children and adults two years after introduction of the thirteen valent pneumococcal conjugate vaccine in England. Vaccine. 2014 10.1016/j.vaccine.2014.03.017 . 24657717

[12] Azzari C, Martinon-Torres F, Schmitt HJ, Dagan R. Evolving Role of 13-Valent Pneumococcal Conjugate Vaccine in Clinical Practice. The Pediatric infectious disease journal. 2014 10.1097/INF.0000000000000328 . 24618937

[13] Blanchard-Rohner G, Pollard AJ. Long-term protection after immunization with protein-polysaccharide conjugate vaccines in infancy. Expert review of vaccines. 2011;10(5):673–84. 10.1586/erv.11.14 . 21604987

[14] McHeyzer-Williams LJ, Malherbe LP, McHeyzer-Williams MG. Checkpoints in memory B-cell evolution. Immunological reviews. 2006;211:255–68. 10.1111/j.0105-2896.2006.00397.x . 16824133

[15] Kelly DF, Moxon ER, Pollard AJ. Haemophilus influenzae type b conjugate vaccines. Immunology. 2004;113(2):163–74. 10.1111/j.1365-2567.2004.01971.x 15379976 PMC1782565

[16] Adderson EE. Antibody repertoires in infants and adults: effects of T-independent and T-dependent immunizations. Springer seminars in immunopathology. 2001;23(4):387–403. . 11826616 10.1007/s281-001-8166-x

[17] Rijkers GT, Sanders EA, Breukels MA, Zegers BJ. Infant B cell responses to polysaccharide determinants. Vaccine. 1998;16(14–15):1396–400. . 9711778 10.1016/s0264-410x(98)00098-x

[18] Kubler-Kielb J, Vinogradov E, Lagergard T, Ginzberg A, King JD, Preston A, et al Oligosaccharide conjugates of Bordetella pertussis and bronchiseptica induce bactericidal antibodies, an addition to pertussis vaccine. Proceedings of the National Academy of Sciences of the United States of America. 2011;108(10):4087–92. 10.1073/pnas.1100782108 21367691 PMC3054038

[19] Poolman J, Frasch C, Nurkka A, Kayhty H, Biemans R, Schuerman L. Impact of the conjugation method on the immunogenicity of Streptococcus pneumoniae serotype 19F polysaccharide in conjugate vaccines. Clinical and vaccine immunology: CVI. 2011;18(2):327–36. 10.1128/CVI.00402-10 21123523 PMC3067356

[20] Vesikari T, Wysocki J, Chevallier B, Karvonen A, Czajka H, Arsene JP, et al Immunogenicity of the 10-valent pneumococcal non-typeable Haemophilus influenzae protein D conjugate vaccine (PHiD-CV) compared to the licensed 7vCRM vaccine. The Pediatric infectious disease journal. 2009;28(4 Suppl):S66–76. Epub 2009/04/11. 10.1097/INF.0b013e318199f8ef . 19325449

[21] Prymula R, Schuerman L. 10-valent pneumococcal nontypeable Haemophilus influenzae PD conjugate vaccine: Synflorix. Expert review of vaccines. 2009;8(11):1479–500. 10.1586/erv.09.113 . 19863240

[22] Grant LR, O'Brien SE, Burbidge P, Haston M, Zancolli M, Cowell L, et al Comparative immunogenicity of 7 and 13-valent pneumococcal conjugate vaccines and the development of functional antibodies to cross-reactive serotypes. PloS one. 2013;8(9):e74906 10.1371/journal.pone.0074906 24086394 PMC3781100

[23] Dagan R, Patterson S, Juergens C, Greenberg D, Givon-Lavi N, Porat N, et al Comparative immunogenicity and efficacy of 13-valent and 7-valent pneumococcal conjugate vaccines in reducing nasopharyngeal colonization: a randomized double-blind trial. Clinical infectious diseases: an official publication of the Infectious Diseases Society of America. 2013;57(7):952–62. 10.1093/cid/cit428 . 23804191

[24] van den Bergh MR, Spijkerman J, Francois N, Swinnen K, Borys D, Schuerman L, et al Immunogenicity, safety, and reactogenicity of the 10-valent pneumococcal nontypeable Haemophilus influenzae protein D conjugate vaccine and DTPa-IPV-Hib when coadministered as a 3-dose primary vaccination schedule in The Netherlands: a randomized controlled trial. The Pediatric infectious disease journal. 2011;30(9):e170–8. 10.1097/INF.0b013e31821a0614 . 21487327

[25] Knuf M, Pankow-Culot H, Grunert D, Rapp M, Panzer F, Kollges R, et al Induction of immunologic memory following primary vaccination with the 10-valent pneumococcal nontypeable Haemophilus influenzae protein D conjugate vaccine in infants. The Pediatric infectious disease journal. 2012;31(1):e31–6. 10.1097/INF.0b013e3182323ac2 . 21909049

[26] Plosker GL. 13-valent pneumococcal conjugate vaccine: a review of its use in infants, children, and adolescents. Paediatric drugs. 2013;15(5):403–23. 10.1007/s40272-013-0047-z . 24030738

[27] van Westen E, Wijmenga-Monsuur AJ, van Dijken HH, van Gaans-van den Brink JA, Kuipers B, Knol MJ, et al Differential B-Cell Memory Around the 11-Month Booster in Children Vaccinated With a 10- or 13-Valent Pneumococcal Conjugate Vaccine. Clinical infectious diseases: an official publication of the Infectious Diseases Society of America. 2015 10.1093/cid/civ274 . 25838290 PMC4503810

[28] Elberse KE, Tcherniaeva I, Berbers GA, Schouls LM. Optimization and application of a multiplex bead-based assay to quantify serotype-specific IgG against Streptococcus pneumoniae polysaccharides: response to the booster vaccine after immunization with the pneumococcal 7-valent conjugate vaccine. Clinical and vaccine immunology: CVI. 2010;17(4):674–82. Epub 2010/02/05. 10.1128/CVI.00408-09 20130129 PMC2849341

[29] Spijkerman J, Veenhoven RH, Wijmenga-Monsuur AJ, Elberse KE, van Gageldonk PG, Knol MJ, et al Immunogenicity of 13-valent pneumococcal conjugate vaccine administered according to 4 different primary immunization schedules in infants: a randomized clinical trial. JAMA: the journal of the American Medical Association. 2013;310(9):930–7. 10.1001/jama.2013.228052 . 24002279

[30] Burton RL, Nahm MH. Development and validation of a fourfold multiplexed opsonization assay (MOPA4) for pneumococcal antibodies. Clinical and vaccine immunology: CVI. 2006;13(9):1004–9. Epub 2006/09/09. 10.1128/CVI.00112-06 16960111 PMC1563573

[31] van Gageldonk PG, van Schaijk FG, van der Klis FR, Berbers GA. Development and validation of a multiplex immunoassay for the simultaneous determination of serum antibodies to Bordetella pertussis, diphtheria and tetanus. Journal of immunological methods. 2008;335(1–2):79–89. Epub 2008/04/15. 10.1016/j.jim.2008.02.018 . 18407287

[32] de Voer RM, Schepp RM, Versteegh FG, van der Klis FR, Berbers GA. Simultaneous detection of Haemophilus influenzae type b polysaccharide-specific antibodies and Neisseria meningitidis serogroup A, C, Y, and W-135 polysaccharide-specific antibodies in a fluorescent-bead-based multiplex immunoassay. Clinical and vaccine immunology: CVI. 2009;16(3):433–6. Epub 2009/01/09. 10.1128/CVI.00364-08 19129470 PMC2650869

[33] van Gageldonk PG, von Hunolstein C, van der Klis FR, Berbers GA. Improved specificity of a multiplex immunoassay for quantitation of anti-diphtheria toxin antibodies with the use of diphtheria toxoid. Clinical and vaccine immunology: CVI. 2011;18(7):1183–6. Epub 2011/05/27. 10.1128/CVI.05081-11 21613460 PMC3147321

[34] Goldblatt D, Southern J, Ashton L, Richmond P, Burbidge P, Tasevska J, et al Immunogenicity and boosting after a reduced number of doses of a pneumococcal conjugate vaccine in infants and toddlers. The Pediatric infectious disease journal. 2006;25(4):312–9. 10.1097/01.inf.0000207483.60267.e7 . 16567982

[35] Omenaca F, Merino JM, Tejedor JC, Constantopoulos A, Papaevangelou V, Kafetzis D, et al Immunization of preterm infants with 10-valent pneumococcal conjugate vaccine. Pediatrics. 2011;128(2):e290–8. 10.1542/peds.2010-1184 . 21727108

[36] Silfverdal SA, Hogh B, Bergsaker MR, Skerlikova H, Lommel P, Borys D, et al Immunogenicity of a 2-dose priming and booster vaccination with the 10-valent pneumococcal nontypeable Haemophilus influenzae protein D conjugate vaccine. The Pediatric infectious disease journal. 2009;28(10):e276–82. 10.1097/INF.0b013e3181b48ca3 . 20118683

[37] Vesikari T, Karvonen A, Lindblad N, Korhonen T, Lommel P, Willems P, et al Safety and immunogenicity of a booster dose of the 10-valent pneumococcal nontypeable Haemophilus influenzae protein D conjugate vaccine coadministered with measles-mumps-rubella-varicella vaccine in children aged 12 to 16 months. The Pediatric infectious disease journal. 2010;29(6):e47–56. 10.1097/INF.0b013e3181dffabf . 20508478

[38] Wysocki J, Tejedor JC, Grunert D, Konior R, Garcia-Sicilia J, Knuf M, et al Immunogenicity of the 10-valent pneumococcal non-typeable Haemophilus influenzae protein D conjugate vaccine (PHiD-CV) when coadministered with different Neisseria meningitidis serogroup C conjugate vaccines. The Pediatric infectious disease journal. 2009;28(4 Suppl):S77–88. 10.1097/INF.0b013e318199f609 . 19325450

[39] Bergh van den MR. Nasopharyngeal colonization with respiratory pathogens. Thesis. 2013.

[40] Siber GR, Chang I, Baker S, Fernsten P, O'Brien KL, Santosham M, et al Estimating the protective concentration of anti-pneumococcal capsular polysaccharide antibodies. Vaccine. 2007;25(19):3816–26. 10.1016/j.vaccine.2007.01.119 . 17368878

[41] Andrews NJ, Waight PA, Burbidge P, Pearce E, Roalfe L, Zancolli M, et al Serotype-specific effectiveness and correlates of protection for the 13-valent pneumococcal conjugate vaccine: a postlicensure indirect cohort study. The Lancet Infectious diseases. 2014;14(9):839–46. 10.1016/S1473-3099(14)70822-9 . 25042756

[42] Park IH, Moore MR, Treanor JJ, Pelton SI, Pilishvili T, Beall B, et al Differential effects of pneumococcal vaccines against serotypes 6A and 6C. The Journal of infectious diseases. 2008;198(12):1818–22. Epub 2008/11/06. 10.1086/593339 . 18983249 PMC4159939

[43] Simell B, Nurkka A, Ekstrom N, Givon-Lavi N, Kayhty H, Dagan R. Serum IgM antibodies contribute to high levels of opsonophagocytic activities in toddlers immunized with a single dose of the 9-valent pneumococcal conjugate vaccine. Clinical and vaccine immunology: CVI. 2012;19(10):1618–23. 10.1128/CVI.00248-12 22875604 PMC3485875

[44] van 't Schurink-van ‘t Klooster TM, de Melker HE, van der Avoort HGAM, van Beers CJAM, Benschop K, G.A.M. Berbers, et al. The National Immunisation Programme; Surveillance and developments in 2013–2014 in the Netherlands. RIVM Report 151103001/2014. 2014.

[45] Domingues CM, Verani JR, Montenegro Renoiner EI, de Cunto Brandileone MC, Flannery B, de Oliveira LH, et al Effectiveness of ten-valent pneumococcal conjugate vaccine against invasive pneumococcal disease in Brazil: a matched case-control study. The Lancet Respiratory medicine. 2014;2(6):464–71. 10.1016/S2213-2600(14)70060-8 . 24726406 PMC9003592

[46] Palmu AA, Jokinen J, Borys D, Nieminen H, Ruokokoski E, Siira L, et al Effectiveness of the ten-valent pneumococcal Haemophilus influenzae protein D conjugate vaccine (PHiD-CV10) against invasive pneumococcal disease: a cluster randomised trial. Lancet. 2013;381(9862):214–22. 10.1016/S0140-6736(12)61854-6 . 23158882

[47] Yeh SH, Gurtman A, Hurley DC, Block SL, Schwartz RH, Patterson S, et al Immunogenicity and safety of 13-valent pneumococcal conjugate vaccine in infants and toddlers. Pediatrics. 2010;126(3):e493–505. 10.1542/peds.2009-3027 . 20732948

[48] Hausdorff WP, Hoet B, Schuerman L. Do pneumococcal conjugate vaccines provide any cross-protection against serotype 19A? BMC pediatrics. 2010;10:4 10.1186/1471-2431-10-4 20122261 PMC2829470

[49] Millar EV, Pimenta FC, Roundtree A, Jackson D, Carvalho Mda G, Perilla MJ, et al Pre- and post-conjugate vaccine epidemiology of pneumococcal serotype 6C invasive disease and carriage within Navajo and White Mountain Apache communities. Clinical infectious diseases: an official publication of the Infectious Diseases Society of America. 2010;51(11):1258–65. 10.1086/657070 . 21034194

[50] Whitney CG, Pilishvili T, Farley MM, Schaffner W, Craig AS, Lynfield R, et al Effectiveness of seven-valent pneumococcal conjugate vaccine against invasive pneumococcal disease: a matched case-control study. Lancet. 2006;368(9546):1495–502. 10.1016/S0140-6736(06)69637-2 . 17071283

[51] Harboe ZB, Dalby T, Weinberger DM, Benfield T, Molbak K, Slotved HC, et al Impact of 13-valent pneumococcal conjugate vaccination in invasive pneumococcal disease incidence and mortality. Clinical infectious diseases: an official publication of the Infectious Diseases Society of America. 2014;59(8):1066–73. 10.1093/cid/ciu524 . 25034421

[52] Kaplan SL, Barson WJ, Lin PL, Romero JR, Bradley JS, Tan TQ, et al Early trends for invasive pneumococcal infections in children after the introduction of the 13-valent pneumococcal conjugate vaccine. The Pediatric infectious disease journal. 2013;32(3):203–7. 10.1097/INF.0b013e318275614b . 23558320

[53] Demczuk WH, Martin I, Griffith A, Lefebvre B, McGeer A, Lovgren M, et al Serotype distribution of invasive Streptococcus pneumoniae in Canada after the introduction of the 13-valent pneumococcal conjugate vaccine, 2010–2012. Canadian journal of microbiology. 2013;59(12):778–88. 10.1139/cjm-2013-0614 . 24313450

[54] Knuf M, Szenborn L, Moro M, Petit C, Bermal N, Bernard L, et al Immunogenicity of routinely used childhood vaccines when coadministered with the 10-valent pneumococcal non-typeable Haemophilus influenzae protein D conjugate vaccine (PHiD-CV). The Pediatric infectious disease journal. 2009;28(4 Suppl):S97–S108. 10.1097/INF.0b013e318199f61b . 19325452

