# Correction: Direct Comparison of Immunogenicity Induced by 10- or 13-Valent Pneumococcal Conjugate Vaccine around the 11-Month Booster in Dutch Infants

**DOI:** 10.1371/journal.pone.0155088

**Published:** 2016-05-11

**Authors:** Alienke J. Wijmenga-Monsuur, Els van Westen, Mirjam J. Knol, Riet M. C. Jongerius, Marta Zancolli, David Goldblatt, Pieter G. M. van Gageldonk, Irina Tcherniaeva, Guy A. M. Berbers, Nynke Y. Rots

The Funding section is incorrect. The correct funding information is as follows: This research was supported by the National Institute for Health Research Biomedical Research Centre at Great Ormond Street Hospital for Children NHS Foundation Trust and University College London.
