# Supplementary material for: Direct Comparison of Immunogenicity Induced by 10- or 13-Valent Pneumococcal Conjugate Vaccine around the 11-Month Booster in Dutch Infants
Source: PLoS One. 2015 Dec 10;10(12):e0144739. doi: 10.1371/journal.pone.0144739 (PMC4690595; doi:10.1371/journal.pone.0144739)
Supplement: S1 Text — (PDF) [file pone.0144739.s001.pdf]

Development of humoral and cellular immune response in infants after pneumococcal conjugate vaccinations with Synflorix® or Prevenar-13®

Includes amendment 1

Protocol No: VAC-259
Version: 4.0
Date: March 16, 2012

Sponsor: National Institute of Health and the Environment (RIVM)
Antonie van Leeuwenhoeklaan 9
PO box 1
3720 BA Bilthoven, The Netherlands
Phone: +31 30 274 3299
Fax: +31 30 274 4415

PROTOCOL TITLE 'Development of humoral and cellular immune response in infants after pneumococcal conjugate vaccinations with Synflorix® or Prevenar-13®'

**Protocol ID**	**VAC-259, NL37173.000.11**
**Short title**	**Pneumococcal immune response to PCV10 and PCV13 in infants**
**Nederlandse leken titel**	**Immuniteit en immunologisch geheugen na pneumokokkenvaccinatie in kinderen**
**Version**	**4.0**
**Date**	**March 16, 2012**
**Principal investigator/project leader**	**RIVM/CIB/VAC/CRR**<br>**Dr AJ Wijmenga-Monsuur**<br>**Dept. Vaccinology-Clinical and Regulatory Research**<br>**National Institute of Health and the Environment (RIVM)**<br>**Antonie van Leeuwenhoeklaan 9**<br>**PO box 1, 3720 BA Bilthoven, The Netherlands**<br>**Internal mailbox 41**<br>**T: +31 302743299**<br>**F: +31 302744415**<br>**E: Alienke.wijmenga@rivm.nl**
**Sponsor (in Dutch: verrichter/opdrachtgever)**	**National Institute of Health and the Environment (RIVM)**
**Institutional partners**	**RIVM/CIB/VAC/VR**<br>**Drs E van Westen, PhD student immunology**<br>**T: +31 302743999**<br>**E: Els.van.Westen@rivm.nl**
	**RIVM/CIB/VAC/CRR**<br>**Dr. MAC de Bruijn, co-projectleader**

	**T: +31 302743693**<br>**E: Marianne.de.Bruijn@rivm.nl**
	**RIVM/CIB/VAC/PM**<br>**Dr NY Rots, Senior clinical affairs scientist, Program Manager Clinical & Immunological**<br>**T: +31 302744080**<br>**E: Nynke.Rots@rivm.nl**
	**RIVM/CIB/LIS**<br>**Dr GAM Berbers, immunologist**<br>**T: +31 302742496**<br>**E: Guy.Berbers@rivm.nl**<br>**Dr LM Schouls, Project leader bacterial meningitis**<br>**T: +31 302742121**<br>**E: Leo.Schouls@rivm.nl**
	**RIVM/CIB/EPI**<br>**Dr HE de Melker, epidemiologist**<br>**T: +31 302743958**<br>**E: Hester.de.Melker@rivm.nl**
	**RIVM/CIB/VAC/CRR**<br>**MC Jongerius, klinisch onderzoeksmedewerker**<br>**T: +31 302743465**<br>**E: Riet.Jongerius@rivm.nl**
**Independent physician(s)**	**Dr LJ Bont, physician children infectious diseases UMCU, Children's Hospital**<br>**T: +31 0887554001**<br>**E: L.Bont@umcutrecht.nl**
**Safety Officer**	**RIVM/CIB**<br>**Drs. H. Van Vliet, MD**

	M: +31 625088416<br>E: Hans.van.Vliet@rivm.nl
Laboratory sites	RIVM/CIB/VAC/VR<br>*Opsonophagocytosis, cellular immunogenicity*<br>Drs E van Westen, PhD student immunology<br>T: +31 302743999<br>E: Els.van.Westen@rivm.nl<br>RIVM/CIB/LIS<br>*Antibody concentrations and avidity*<br>Dr GAM Berbers, immunologist<br>T: +31 302742496<br>E: Guy.Berbers@rivm.nl

PROTOCOL SIGNATURE SHEET

Name	Signature	Date
**Sponsor or legal representative:**<br>***National Institute for Public Health and the Environment,***<br>***Centre for Infectious Disease Control (CIb)***<br>**For non-commercial research,**<br>**Head of Division:**<br>***Prof. Dr. R.A. Coutinho***	**Not needed**	
**Dr NY Rots**<br>**Program Manager Clinical & Immunological**<br>**Senior Clinical Affairs Scientist**<br>**RIVM/CIB/VAC/PM**		19 March 2012
**Dr AJ Wijmenga-Monsuur**<br>**Principal investigator**<br>**Project Leader Pneumococcal Vaccines**<br>**Clinical Affairs Scientist**<br>**Author of protocol**<br>**RIVM/CIB/VAC/CRR**		19 march 2012
**Dr GAM Berbers**<br>**Immunologist and project leader of the PIM study**<br>**RIVM/CIB/LIS**		19/03/2012

TABLE OF CONTENTS

1. INTRODUCTION AND RATIONALE 13
2. OBJECTIVES 18
3. STUDY DESIGN 19
4. STUDY POPULATION 22
   4.1 Population (base) 22
   4.2 Inclusion criteria 22
   4.3 Exclusion criteria 22
   4.4 Sample size calculation 23
5. TREATMENT OF SUBJECTS 25
   5.1 Investigational product/treatment 25
   5.2 Use of co-intervention (if applicable) 25
   5.3 Escape medication (if applicable) 25
6. INVESTIGATIONAL MEDICINAL PRODUCT 26
   6.1 Name and description of investigational medicinal product(s) 26
   6.2 Summary of findings from non-clinical studies 26
   6.3 Summary of findings from clinical studies 26
   6.4 Summary of known and potential risks and benefits 26
   6.5 Description and justification of route of administration and dosage 26
   6.6 Dosages, dosage modifications and method of administration 27
   6.7 Preparation and labelling of Investigational Medicinal Product 27
   6.8 Drug accountability 27
7. METHODS 28
   7.1 Study parameters/endpoints 28
      7.1.1 Main study parameter/endpoint 28
      7.1.2 Secondary study parameters/endpoints 28
      7.1.3 Other study parameters 28
   7.2 Randomisation, blinding and treatment allocation 28
   7.3 Study procedures 29
   7.4 Withdrawal of individual subjects 31
   7.5 Replacement of individual subjects after withdrawal 32
   7.6 Follow-up of subjects withdrawn from treatment 32
   7.7 Premature termination of the study 32
8. SAFETY REPORTING 33
   8.1 Section 10 WMO event 33
   8.2 Adverse and serious adverse events 33
      8.2.1 Suspected unexpected serious adverse reactions (SUSAR) 34
      8.2.2 Annual safety report 35
   8.3 Follow-up of adverse events 35
   8.4 Data Safety Monitoring Board (DSMB) 35
9. STATISTICAL ANALYSIS 36
   9.1 Descriptive statistics 36

9.2 Univariate analysis ... 36
9.3 Multivariate analysis ... 37
10. ETHICAL CONSIDERATIONS ... 38
10.1 Regulation statement ... 38
10.2 Recruitment and consent ... 38
10.3 Objection by minors or incapacitated subjects (if applicable) ... 38
10.4 Benefits and risks assessment, group relatedness ... 38
10.5 Compensation for injury ... 39
10.6 Incentives ... 40
11. ADMINISTRATIVE ASPECTS AND PUBLICATION ... 41
11.1 Handling and storage of data and documents ... 41
11.2 Amendments ... 41
11.3 Annual progress report ... 41
11.4 End of study report ... 42
11.5 Public disclosure and publication policy ... 42
12. REFERENCES ... 43

LIST OF ABBREVIATIONS AND RELEVANT DEFINITIONS

ABR	ABR form, General Assessment and Registration form, is the application form that is required for submission to the accredited Ethics Committee (In Dutch, ABR = Algemene Beoordeling en Registratie)
AE	Adverse Event
AR	Adverse Reaction
CA	Competent Authority
CCMO	Central Committee on Research Involving Human Subjects; in Dutch: Centrale Commissie Mensgebonden Onderzoek
CV	Curriculum Vitae
DSMB	Data Safety Monitoring Board
EU	European Union
EudraCT	European drug regulatory affairs Clinical Trials
GCP	Good Clinical Practice
HC	Health Council
IB	Investigator’s Brochure
IC	Informed Consent
IMP	Investigational Medicinal Product
IMPD	Investigational Medicinal Product Dossier
IPD	Invasive Pneumococcal Disease
METC	Medical research ethics committee (MREC); in Dutch: medisch ethische toetsing commissie (METC)
NIP	Netherlands Immunization Program
NP	Nasopharyngeal
PCV	Pneumococcal Conjugate Vaccine
PCV7	Prevenar®, 7-valent pneumococcal conjugate vaccine (Phizer/Wyeth)
PCV10	Synflorix®, 10-valent pneumococcal conjugate vaccine (GSK)
PCV13	Prevenar-13®, 13-valent pneumococcal conjugate vaccine (Phizer/Wyeth)
(S)AE	(Serious) Adverse Event
SP	*Streptococcus pneumonia*
SPC	Summary of Product Characteristics (in Dutch: officiële productinfomatie IB1-tekst)
Sponsor	The sponsor is the party that commissions the organisation or performance of the research, for example a pharmaceutical company, academic hospital, scientific organisation or investigator. A party that provides funding for a study but does not commission it is not regarded as the sponsor, but

	**referred to as a subsidising party.**
**SUSAR**	**Suspected Unexpected Serious Adverse Reaction**
**Wbp**	**Personal Data Protection Act (in Dutch: Wet Bescherming Persoonsgevens)**
**WMO**	**Medical Research Involving Human Subjects Act (in Dutch: Wet Medisch-wetenschappelijk Onderzoek met Mensen**

SUMMARY

Rationale:

Streptococcus pneumonia (SP) is an important cause of morbidity and mortality worldwide, with the highest incidence of disease among children < 2 years of age. *Streptococcus pneumonia* consisting of > 90 known different serotypes, of which a limited number of about 20 serotypes are known to cause invasive pneumococcal disease (IPD).

Prevenar®, a seven-valent pneumococcal conjugate vaccine (PCV7) was first introduced in the Netherlands immunization program (NIP) for children born after April 2006. It confers protection against serotypes 4, 6B, 9V, 14, 18C, 19F and 23F (see figure 1). It has been introduced in the NIP for vaccination at 2, 3, 4 and 11 months of age. Recently in 2009, two new vaccines were registered, which can in due time replace PCV7 in the NIP. All children born after March 2011 will receive Synflorix®, a ten-valent pneumococcal conjugate vaccine (PCV10) which confers protection against three additional serotypes. Prevenar-13®, a thirteen-valent pneumococcal conjugate vaccine (PCV13) confers protection against another three extra serotypes, but is not implemented in the NIP.

The current study in combination with our previous KOKKI (cellular immunogenicity after PCV7 vaccination) and PIM (comparison of 4 different PCV13 vaccination schedules based on humoral immunogenicity) study can give input to the Health Council (HC) on the best vaccination strategy for pneumococcal vaccination. The outcomes of this trial will provide data on the humoral and cellular immune response of PCV10 and PCV13.

Objective:
Primary: To compare immunogenicity (humoral and cellular) induced by PCV10 and PCV13 after the booster dose of a complete vaccination series (3+1, the current NIP schedule)

Secondary:
To compare immunogenicity (humoral) induced by PCV10 and PCV13 at 5, 8, 11 and 12 months of a complete vaccination series (3+1, the current NIP schedule)

To investigate the possible influence of the pneumococcal vaccination on the serological responses of other vaccine components of the NIP which are administered simultaneously in the other limb (DTaP-Hib)

Study design:

A controlled randomized intervention trial with 2 groups (see figure 2 and table 1).

- Group 1 PCV13
  - Divided in group 1a and 1b; 33 infants per group

- Group 2 PCV10
  - Divided in group 2a and 2b; 33 infants per group

Group 1 and 2 are split in sub groups in order to reduce the burden of the 8 ml blood samples. Randomization will be done within group 1 and within group 2.

Study population:

Children eligible to receiving the regular vaccinations of the NIP, born after August 2011 (assuring that all children are eligible for and will receive Hepatitis B vaccination)

Intervention:

Children of group 1 will receive the DTaP-IPV-Hib-HepB vaccination according to the NIP; they will receive PCV13 instead of PCV10. All vaccinations will be given by the study team during home visits.

Children of group 2 will receive all vaccinations (DTaP-IPV-Hib-HepB and PCV10) as part of the NIP by a well-baby clinic nurse; this is not part of the trial.

Main study parameters/endpoints:

Primary

Pneumococcal serotypes

- Cellular immune response (Plasma B cells and memory B cells) immediately before and 7-9 days after the booster at 11-months of age
- Humoral immune response (antibody concentrations and geometric mean concentrations (GMT)) at 12 months of age

Secondary

Pneumococcal serotypes

- Opsonophagocytoses immediately before and 7-9 days after the booster at 11-months of age
- Avidity at 5, 8, immediately before and 7-9 days after the booster at 11-months and at 12 months of age

- Antibody concentrations and geometric mean concentrations (GMT) at 5, 8, immediately before and 7-9 days after the booster at 11-months and at 12 months of age
- Kinetics of antibody concentrations and geometric mean concentrations (GMT) over time (at 5, 8, 11 and 12 months of age)

DTaP-HIb

- Antibody concentrations and geometric mean concentrations (GMT) at 5, 8, 11 and 12 months of age

Nature and extent of the burden and risks associated with participation, benefit and group relatedness:

One blood collection of 8 ml (2x 4 ml tubes). The burden and risk is considered low.
The children might find the needle scary and it might be painful but only for a few seconds. A local anaesthetic (Emla® crème, Astra Zeneca) may be used to minimize pain. Blood collection could result in a small bruise at the location of injection, which will disappear within a few days.
Group 1; one heel/finger stick sampling, group 2: 3-4 heel/finger sticks sampling. The burden and risk is considered low.

For group 2 (PCV10 group), the children themselves have no direct benefit in participating in this trial. The trial is aimed to study the immune response after 3+1 PCV10 or PCV13 vaccinations. These children, who have followed the Dutch NIP, are the only possible children that can participate in the trial. Visits will take 10-30 minutes each (depending on the type of blood collection and whether a questionnaire is taken).

Children in group 1 will receive PCV13 vaccinations. The side effects of these vaccinations are expected to be equal to the side effects of PCV10 (which the children would have received as part of the NIP). They will however receive these vaccinations at home to reduce the burden. These children will benefit from the added protection of the three extra serotypes which are not present in the PCV10 vaccination. These children are the only possible study group, since they are eligible for the Dutch NIP. Visits will take maximum 30 minutes each.

1. INTRODUCTION AND RATIONALE

Streptococcus pneumonia (SP) is an important cause of morbidity and mortality worldwide, with the highest incidence of disease among children < 2 years of age. *Streptococcus pneumonia* consisting of > 90 known different serotypes, of which a limited number of about 20 serotypes are known to cause invasive pneumococcal disease (IPD) such as bacteraemia, sepsis and meningitis, bone and joint infections or respiratory infections like pneumonia and otitis media (1). IPD is always preceded by nasopharyngeal (NP) pneumococcal colonization. In industrialised countries like the Netherlands, NP pneumococcal colonisation increases to 50% or more during the first year of life and remains high until the age of 3 years after which it decreases to a stable colonisation of 10-20% after 10 years of age (2, 3). NP carriage of infants and young children is an important source of organism transmission. In most cases pneumococcal carriers remain asymptomatic but they can develop severe airway infections or invasive disease.

As of June 2006 Pneumococcal Conjugate Vaccination (PCV) has been added to the Netherlands Immunization Program (NIP) for children born after April 2006. Prevenar®, a seven-valent pneumococcal conjugate vaccine (PCV7), was the first registered vaccine. It confers protection against serotypes 4, 6B, 9V, 14, 18C, 19F and 23F (see figure 1). It has been introduced in the NIP for vaccination at 2, 3, 4 and 11 months of age. Around 50% of European countries who use a PCV vaccine have introduced the vaccine in a 2+1 vaccination schedule (e.g. 2, 4 and 11 months of age or 3, 5 and 12 months of age). Recently in 2009, two new vaccines were registered, which can in due time replace PCV7 in the NIP. Synflorix®, a ten-valent pneumococcal conjugate vaccine (PCV10) confers protection against three additional serotypes (see figure 1). Prevenar-13®, a thirteen-valent pneumococcal conjugate vaccine (PCV13) confers protection against another three additional serotypes (see figure 1). Besides increasing serotype coverage of the three PCV vaccines, also the conjugate is different. Most* serotypes of PCV10 are conjugated to protein D of non-typeable Haemophilus influenzae, instead of CRM197 carrier protein (conjugate for PCV7 and PCV13), which might influence the induction of memory. *(*except for type 18C who is conjugated to tetanus toxoid carrier protein and type 19F to diphtheria toxoid carrier protein)*

As of March 2011 all newborns will receive PCV10 vaccinations instead of PCV7.

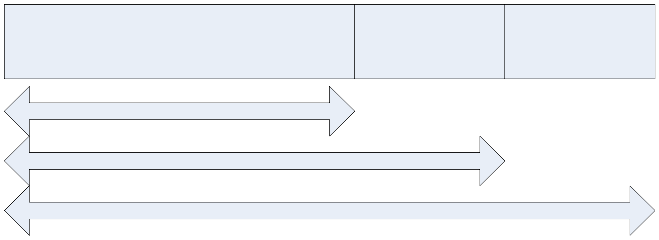

Figure 1 Serotypes present in the three pneumococcal conjugate vaccines

When comparing pneumococcal vaccines an IgG antibody concentration of ≥0.35 ug/ml is used according to the recommendations of the WHO expert committee on biological standardization (4). The antibody concentrations are generally measured at one month after the complete series.

PCV13 showed to be non-inferior to PCV7 for 6 out of 7 serotypes present in PCV7 (except for 6B), when comparing proportions of responders (≥0.35 ug/ml) and geometric mean concentrations (see SPC of PCV13 and (5)). In addition it was shown that PCV13 elicits OPA responses comparable to those elicited by PCV7.
PCV10 induces slightly lower antibody concentrations then PCV7 but are similar in terms of % responders (see SPC of PCV10 and (6)).

PCVs are thought to induce immediate protection through stimulation of antibody production by polysaccharide specific plasma B cells, sustained protection is conferred by a memory B cell pool which induces an accelerated increase in antibody concentrations during secondary immune responses seen after re-infection or boosting. Both the induction and maintenance of functional serum antibody concentrations have a cellular basis which is still poorly understood.

In general, antigens trigger naïve B-cells to expand and differentiate into two types of affinity matured B-cells: antibody secreting plasma B cells and memory B-cells. This immunological B cell priming occurs in highly organized structures of lymphoid tissues, under the influence of cognate CD4+ T cell help. Since plasma cells are unlikely to persist for more than 6-8 weeks (7) maintenance of steady state antibody levels over periods of years requires a continuous low level of differentiation of memory B cells into plasma cells. Factors such as recurrent antigen exposure for example through carriage might be involved in this process.

Carriage of the 7 serotypes present in PCV7 has decreased since the introduction of PCV in the NIP. This can influence the induction of memory and plasma B-cells, since there is hardly any natural boostering. Some of the extra serotypes present in PCV10 and PCV13 are still influenced by the natural boosters.

General aim of our pneumococcal projects is to determine the most optimal pneumococcal vaccination program for the NIP

The number of vaccinations (2+1 versus 3+1), timing of vaccinations and the product choice depend on several factors such as;

- Humoral immunity, which is determined by the measurement of antibody response
  - Protection in the period of peak incidence of IPD (~8 months of age)
  - Protection after the booster
  - Kinetics of antibody concentrations over time
  - Percentage of antibody concentrations above the correlate of protection threshold
- Cellular immunity, which is determined by the measurement of plasma and memory B-cells, opsonophagocytoses and avidity
  - The vaccination schedule should lead to a sustained protection
  - Decrease in carriage (natural boostering) might lead to lower memory responses
  - Different conjugates can lead to differences in memory responses
    - PCV13 is an extended version of the PCV7 vaccine, 6 serotypes are added and both vaccines are conjugated to CRM197 carrier protein
    - PCV10 has a different conjugate, protein D of non-typeable Haemophilus influenzae
- Vaccine composition
  - Coverage of the products, especially in the Dutch population; PCV13 confers protection to more serotypes then PCV10

KOKKI, PIM and our current study

An improved understanding of the immune biology of the conjugate vaccines, such as PCV10 and PCV13, is essential to develop the best immunization strategies that provide sustained protection. The results from the cellular immunity and the antibody concentrations will help to decide on the best vaccination strategy for pneumococcal vaccination in the NIP. The current study in combination with our previous KOKKI and PIM studies will provide more information for the optimization of the pneumococcal immunization program.

Cellular immunity

The aim of **our previous study** (KOKKI, cellular immunogenicity after PCV7 vaccination (NL24329.000.08), 2009) was to determine the development of the cellular immune response (plasma B cells and memory B-cells) for the 3+1 vaccination schedule of PCV7. Since 2009 several factors have changed:

- PCV10 has been introduced in the NIP (instead of PCV7)
- PCV13 was registered and might become part of the NIP in the future

- Carriage of the serotypes present in PCV7 have decreased in the five years following introduction of PCV7 in the NIP (2006), leading to loss of natural booster, and possibly lower memory responses

We lack information on the memory response to PCV10 and PCV13, therefore **our current study** aims to determine the development of the cellular immune response (plasma B cells and memory B-cells) for the 3+1 vaccination schedule of PCV10 and PCV13 immediately before and 7-9 days after the booster at 11-months of age. Time points of blood collection are similar to those in the previous KOKKI study. Also the antibody concentrations, avidity and opsonophagocytoses will be determined at these time points. These vaccines differ in number of serotypes and type of conjugate and could therefore differ in their cellular immunity.
A selection of 5-6 serotypes will be made; two of the four serotypes tested in the KOKKI study and three to four serotypes added by PCV10 and PCV13 for the current study. Selection will depend on serotype circulation just before analyses.

Humoral immunity

The aim of **our previous study** (PIM, (NL28918.000.09), 2010-2012), was to asses the optimal PCV vaccination schedule for PCV13, based on humoral immunogenicity after four different vaccination schedules.
An alternative timing and reduction of the number of vaccination doses on the serological response directed against the different serotypes of pneumococci was compared to the currently used* 3+1 vaccination schedule. The schedules were two 2+1 vaccination schedule (2, 4 and 11 months of age; 3, 5 and 11 months of age) and two 3+1 vaccination schedules (*2, 3, 4 and 11 months of age; 2, 4, 6 and 11 months of age).
After the start of the study PCV10 was introduced in the NIP and not PCV13. It is therefore important to compare the vaccines for the currently used 3+1 vaccination schedule. Also universal vaccination against Hepatitis B will be introduced for children born after August 2011, and therefore the DTaP-IPV-Hib vaccine will be replaced by DTaP-IPV-Hib-HepB, which could influence the response to PCV13.
The current study will investigate the humoral immunogenicity of PCV10 after the currently used 3+1 vaccination schedule (vaccination at 2, 3, 4 and 11 months of age). The data will be compared to the 3+1 vaccination schedule of PCV13 (PIM study). To rule out the influence of the shift from DTaP-IPV-Hib to DTaP-IPV-Hib-HepB vaccine we will use data from group 1 (PCV13) of the current study, to bridge both studies.
As in the PIM study blood samples are collected at one month after the primary series (5 months), at 8, 11 and 12 months of age. The 12 months sample is used to compare the schedules (primary endpoint). The 8 month sample is chosen, since the peak incidence of

IPD is around 8 months. The 5, 8, 11 and 12 month time points are used to asses the kinetics of antibody concentrations in time. To look for interference of concurrent vaccinations DTaP-Hib will be tested.

Hypothesis used for this study:
PCV10 and PCV13 are comparable in inducing cellular and humoral immune responses in Dutch infants. Differences are expected between serotypes; the 7 serotypes of PCV7 are not under influence of natural boosting; the new serotypes present in the vaccines are still under influence of natural boosting.

2. OBJECTIVES

Primary: To compare immunogenicity (humoral and cellular) induced by PCV10 and PCV13 after the booster dose of a complete vaccination series (3+1, the current NIP schedule)

Secondary:
To compare immunogenicity (humoral) induced by PCV10 and PCV13 at 5, 8, 11 and 12 months of a complete vaccination series (3+1, the current NIP schedule)

To investigate the possible influence of the pneumococcal vaccination on the serological responses of other vaccine components of the NIP which are administered simultaneously in the other limb (DTaP-Hib)

3. STUDY DESIGN

Children eligible to receiving the regular vaccinations of the NIP, born after August 2011 (assuring that all children are eligible for and will receive Hepatitis B vaccination)

A controlled, randomized, intervention trial with 2 groups (see figure 2 and table 1).

- Children of group 1 will be invited first (around the age of 1 month), since they have to receive all DTaP-IPV-Hib-HepB and PCV13 vaccinations as part of the trial
  - Vaccinations will be given during home visits
  - Children will be randomized over 2 sub groups in order to diminish the burden of the 8 ml blood sample (which is collected just before or 7-9 days after the 11-months vaccination)
  - For the immune memory, collect 33 children per sub group in order to have 25 evaluable children per sub group (sub group 1a and 1b)

- Children of group 2 will be invited around the age of 3.5 months, as to include them in the trial at the age of 5 months
  - Vaccinations are not part of the trial, since they already receive them during well-baby clinic visits
  - Children will be randomized over 2 sub groups in order to diminish the burden of the 8 ml blood sample (which is collected just before or 7-9 days after the 11-months vaccination)
  - For the immune memory, collect 33 children per sub group in order to have 25 evaluable children per sub group (sub group 2a and 2b)

Randomization will be done within group 1 and separately within group 2.
The randomization will not be done for the total study (group 1 and 2 together) since children of the PCV10 group already receive all proper vaccinations in the NIP and randomization would result in a long period between invitation at 1 month of age and inclusion at 5 months of age for the PCV10 group.

Figure 2 Study schedule

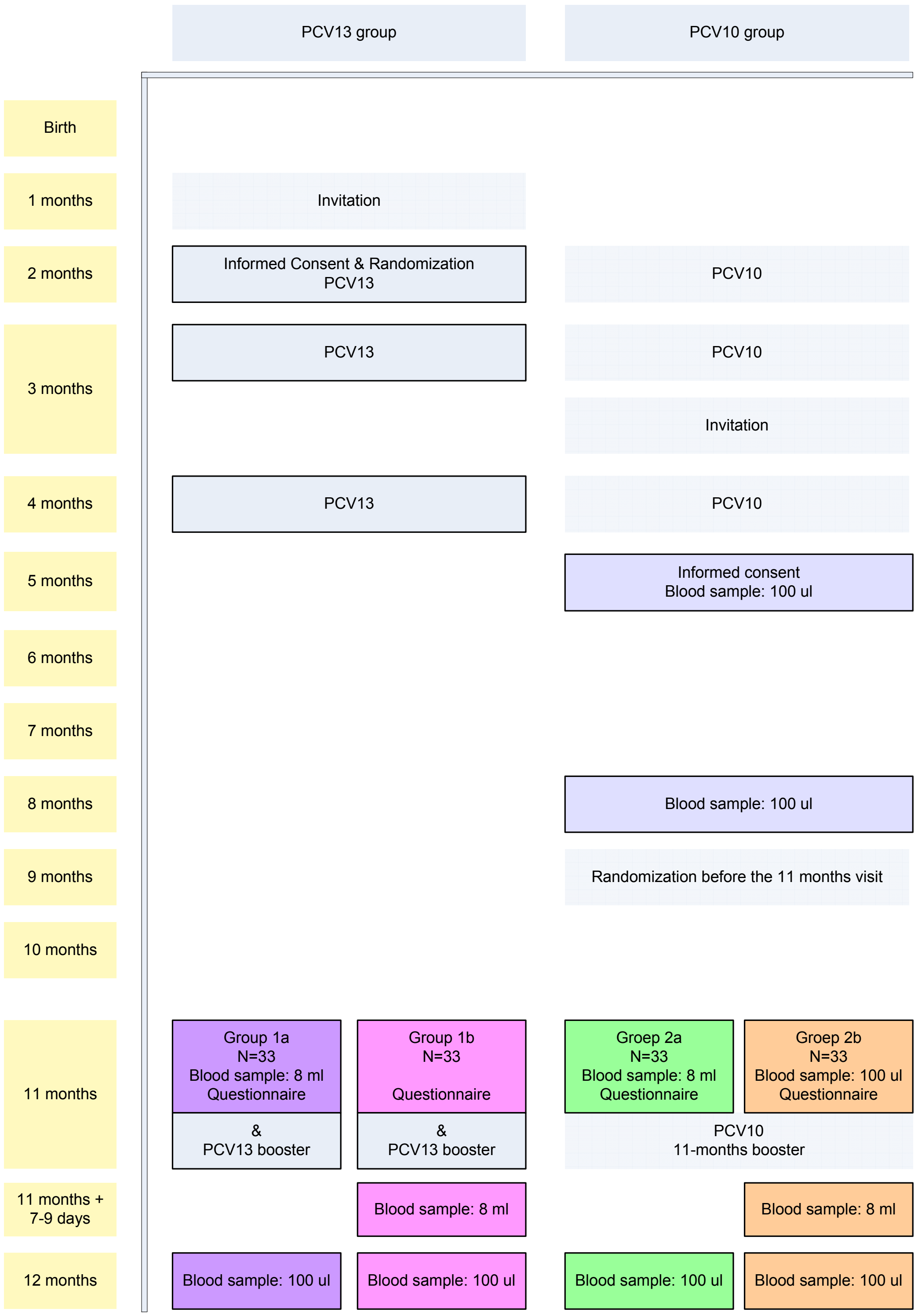

Blood samples (8 ml) are used for cellular immunogenicity (and 100 ul, of these samples will be used for humoral immunogenicity). Blood samples (100 ul) are used for humoral immunogenicity.

	Group 1a (PCV13)	Group 1b (PCV13)	Group 2a (PCV10)	Group 2b (PCV10)
Invitation (~September 2011)	x	x		
Invitation (~Januari 2012)			x	x
Receive response card	x	x	x	x
Send extra information by email	x	x	x	x
Telephone call 1 (inquire about the parents interest, explain the study if necessary, make an appointment)	x	x	x	x
Send the extra information also on paper	x	x	x	x
Check with RCP if child is allowed to receive vaccinations	x	x		
2 months vaccination (PCV13 and DTaP-IPV-Hib-HepB) Including check in- exclusion criteria, signing of IC and randomization	x	x		
3 months vaccination (PCV13 and DTaP-IPV-Hib-HepB)	x	x		
4 months vaccination (PCV13 and DTaP-IPV-Hib-HepB)	x	x		
5 months blood collection (heel/finger stick) Including check in- exclusion criteria, signing of IC			x	x
8 months blood collection (heel/finger stick)			x	x
Randomization			x	x
Pre 11 months blood collection (8 ml) Questionnaire			x	
Pre 11 months blood collection (heel/finger stick) Questionnaire				x
Pre 11 months blood collection (8 ml) & vaccination (PCV13 and DTaP-IPV-Hib-HepB) Questionnaire	x			
Vaccination (PCV13 and DTaP-IPV-Hib-HepB) Questionnaire		x		
Post 11 months blood collection (7-9 days) Questionnaire		x		x
12 months blood collection (heel/finger stick)	x	x	x	x
End of study for participants	x	X	x	x
Deliver all samples at the RIVM (after each blood collection visit day)	x	x	x	x

Table 1 Events during study

4. STUDY POPULATION

4.1 Population (base)

Children eligible to receiving the regular vaccinations of the NIP, born after August 2011 (assuring that all children are eligible for and will receive Hepatitis B vaccination)

4.2 Inclusion criteria

- The children have to be of normal health (same health criteria apply as used in well-baby clinics when a child receives a vaccination, e.g. also children with small increases in temperature (≤38.5 °C) or cold are seen as children with normal health)
- The parents/legally representatives have to be willing and able to allow their child to participate in the trial according to the described procedures
- Presence of a signed informed consent (the parents/legally representatives have given written informed consent after receiving oral and written information)
- Group 1: The children are 2 months old (± 2 weeks), have not received any vaccinations and will receive all vaccinations (DTaP-IPV-Hib-HepB and PCV13) by the study team
- Group 2: The children are 4-6 months old and have received three DTaP-IPV-Hib-HepB and PCV10 vaccinations according to the 3+1 schedule of the Dutch NIP*.

**The Dutch NIP 3+1 schedule: All children born as of August 1st 2011 will receive Synflorix (PCV10) and DTaP-IPV-Hib-HepB vaccinations, at the age of 2, 3 4 and 11 months of age.*

4.3 Exclusion criteria

Temporarily exclusion criteria for group 1

- Children that are ill or have a moderate or high fever (rectal temperature of > 38.5 °C). Vaccination will be postponed until the symptoms of illness and the fever have disappeared

Exclusion criteria

- Group 1: Previous vaccinations with PCV7 or PCV10
- Group 2: Previous vaccinations with PCV7 of PCV13
- Vaccinations using a schedule that differs from the Dutch 3+1 schedule
- Presence of a serious disease that requires medical care that can interfere with the results of the study

- Known or expected allergy/hypersensitivity against one of the vaccine ingredients (anamnestic, be alert if the child has had medical complaints after previous pneumococcal vaccinations)
- Known or suspected immunological disorder
- Previously administration of plasma products (including immunoglobulin), within three months of study enrolment
- Presence of bleeding disorders
- Communication problems that interfere with the trial
- Prematurity (<37 weeks after gestation)

4.4 Sample size calculation

For **cellular immunity**: each group will contain 25 evaluable children (based on previous experience we expect to include at least 30 children in order to reach 25 evaluable children). The group size is based on the previous KOKKI study (NL24329.000.08).

> *'The clinical meaning of differences between the numbers of memory B-cells is still unknown. Therefore power calculations are at the moment not possible. Previous research has shown that the number of memory-B cells circulating in blood is very low before a booster vaccination and in order to observe a trend after revaccination a group of 22 children is needed (including a possible 10% loss of subjects' leads to 25 children for each group).'*

In the KOKKI study, for each child blood was collected at one time point only, being either pre- or 7-9 days post-booster. Inclusion and blood collection was during the same visit. Inclusion in the current study however starts at first vaccination, respectively 9 (group 1) and 6 (group 2) months prior to blood collection for cellular immunity, which can lead to a higher amount of dropout. Taking into account a 10% dropout up to 11 months, we included 33 children in each group. A subsequent estimated failure rate to collect sufficient blood cells of 15% will generate 25 evaluable children.

Antibody concentrations will be compared between the PCV10 group of the current study and the PCV13 group of the PIM study (vaccination schedule 2, 3, 4 and 11 months) at 12 months after vaccination. As significance level we take 0.05 two-sided and as power 80%. The expected variance of log(GMC) is 0.27 (this is for serotype 6B, the serotype with the highest variance). Using the following formula $n = 2k^2\sigma^2 / \delta^2$, we need 47 children per group to detect a 2-fold difference in GMC (=0.30 difference in log(GMC)) between the two groups. A 2- to 2.5-fold difference is considered to represent a true difference in immunogenicity in this type of study (8,9). To account for 20% dropout, 59

children need to be included per group. As we already include 2x33 = 66 children in the cellular immunity part of the study, this group is also large enough to assess antibody concentrations.

The antibody concentrations will be compared for each of the serotypes included in the vaccine. Adjustment for multiple testing is not necessary for these comparisons because the serotypes will not be compared to each other and we are not interested in the overall null hypothesis of no effect (10).

Antibody concentrations will also be compared between the PCV13 group of the current study and the PIM study at 12 months after vaccination to bridge the two studies. Using the same sample size calculation as above, we need 59 children in this group as well.

Based on previous experience we expect a participation rate of 5% for all groups.

The study burden for group 1 involves only one 8 ml blood collection, vaccinations and one heel/finger stick (100 ul) Vaccinations are given at home, which parents appreciate. Since PCV13 is a registered vaccine and is not expected to give more side effects as PCV10, we assume that the study burden is perceived as relatively low, especially when the additional protection of three serotypes is taken into consideration.

The study burden for group 2 involves an 8 ml blood collection and 3-4 heel/finger sticks samples. Although the participants have no benefit in this trial, we expect that the heel/finger stick samples will not be perceived as a high burden.

Due to the lack of benefit for the children in group 2, recruitment rates after the first mailings amounted to around 2% instead of 5%.

In order to reach enough inclusions we need to invite approximately 1200 children for group 1 and 3500 children for group 2. We plan to enroll the children of group 1 in one month and the children of group 2 in 3.5 months.

The RCP region Noord Holland - Utrecht will be used and we will asses the exact region based on data from the Dutch Central Bureau for the Statistics (http://www.cbs.nl), in the same period of 2010. Due to a hampering recruitment for group 2, the RCP region Noord-Holland-Utrecht recruitment area will be extended with parts of Gelderland and Flevoland.

5. TREATMENT OF SUBJECTS

All children in group 2 follow the standard NIP (they have or will receive vaccinations with Synflorix®, a ten-valent pneumococcal conjugate vaccine (PCV10) and DTaP-IPV-Hib-HepB, at the age of 2, 3, 4 and 11 months), which is not part of this study.

5.1 Investigational product/treatment

All children in group 1 will receive Prevenar-13® instead of PCV10. For the rest they will follow the standard NIP, at the age of 2, 3, 4 and 11 months. All vaccinations (PCV13 and DTaP-IPV-Hib-HepB) are given by the study team in order to prevent vaccine mix-up during well-baby clinic appointments. The DTaP-IPV-Hib-HepB is not part of this study, but will be given by the study team for practical reasons.

There is no placebo group present in this trial.

5.2 Use of co-intervention (if applicable)

There is no objection against the use of co-medication or other kinds of interventions against concomitant disorders. The use of painkillers against local pain after vaccination is also not prohibited. Use of immunosuppressive medicines during the trial could in certain cases lead to exclusion of the corresponding blood samples for further laboratory analysis, since that might interfere with the outcomes of the study. Medicine use will be recorded in the questionnaire and the CRF.

5.3 Escape medication (if applicable)

For blood collection a local anesthetic (Emla® crème; AstraZeneca) will be used to minimize the pain.

For vaccination normal NIP practices will be followed.

6. INVESTIGATIONAL MEDICINAL PRODUCT

In this trial **Prevenar-13®**, a thirteen-valent pneumococcal vaccine (PCV13), is used.

Synflorix®, a ten-valent pneumococcal vaccine (PCV10) is not administered in the trial, since children receive this vaccine as part of the NIP. However, we intent to look at the outcome of these vaccinations and therefore we included the information of PCV10 in chapter 6.1-6.4.

6.1 Name and description of investigational medicinal product(s)

PCV13: For the qualitative and quantitative composition see chapter 2 and 6.1 of the SPC.

PCV10: For the qualitative and quantitative composition see chapter 2 and 6.1 of the SPC.

6.2 Summary of findings from non-clinical studies

PCV13: See chapter 5.3, page 12 of the SPC.

PCV10: See chapter 5.3, page 9 of the SPC.

6.3 Summary of findings from clinical studies

PCV13: See chapter 5.1, page 7-11 of the SPC.

PCV10: See chapter 5.1, page 7-9 of the SPC.

6.4 Summary of known and potential risks and benefits

PCV13: See chapter 4 and 5, page 2-12 of the SPC.

PCV10: See chapter 4 and 5, page 2-9 of the SPC.

6.5 Description and justification of route of administration and dosage

PCV13: The vaccine with a dosage of 0.5 ml will be administered i.m., as prescribed by the supplier (Wyeth).

6.6 Dosages, dosage modifications and method of administration

PCV13: The dosage of use is 0.5 ml.
During storage, a white deposit and clear supernatant can be observed. The vaccine should be shaken well to obtain a homogeneous white suspension prior to expelling air from the syringe, and should be inspected visually for any particulate matter and/or variation of physical aspect prior to administration. Do not use if the content appears otherwise.

6.7 Preparation and labelling of Investigational Medicinal Product

PCV13: The normal packaged vials contain 0.5 ml vaccine, as supplied by the manufacturer Wyeth. The vaccine is obtained from Wyeth by the RIVM (RCP/IOD). The vaccine should be withdrawn from the vial lege artis into the syringe for i.m. injection.

6.8 Drug accountability

PCV13: The appropriate number of vaccines are received from the RIVM (RCP/IOD) and stored in a dedicated study fridge. The transfer from the RCP/IOD to the study team will be documented.
All vaccines taken from the fridge by the study team will be documented. The investigator/study team member is responsible for the correct transport and storage conditions to the location of vaccination. The vaccines are transported in isolated coolers that demonstrate the adequate temperature. The vaccine may not be frozen.

In case of unusable vaccines, e.g. vials that are expired or damaged, the vaccine will be returned to the RIVM (RCP/IOD) and replaced.

Each vaccine delivery has to be accompanied with signed receipt form containing information about quantity, expiry date and batch numbers of the supplied vaccines. The form needs to be dated and signed by the person responsible for the transport and the person receiving the product. The investigator is responsible for the accountability in the fridge. If discrepancies are observed between the number of delivered and used vaccines at the end of the study, a written declaration has to be supplied by the investigator. All unused vaccines should be returned to the RIVM (RCP/IOD) at the end of the study.

7. METHODS

7.1 Study parameters/endpoints

7.1.1 Main study parameter/endpoint

Pneumococcal serotypes

- Cellular immune response (Plasma B cells and memory B cells) immediately before and 7-9 days after the booster at 11-months of age
- Humoral immune response (antibody concentrations and geometric mean concentrations (GMT)) at 12 months of age

7.1.2 Secondary study parameters/endpoints

Pneumococcal serotypes

- Opsonophagocytoses immediately before and 7-9 days after the booster at 11-months of age
- Avidity at 5, 8, immediately before and 7-9 days after the booster at 11-months and at 12 months of age
- Antibody concentrations and geometric mean concentrations (GMT) at 5, 8, immediately before and 7-9 days after the booster at 11-months and at 12 months of age
- Kinetics of antibody concentrations and geometric mean concentrations (GMT) over time (at 5, 8, 11 and 12 months of age)

DTaP-HIb

- Antibody concentrations and geometric mean concentrations (GMT) at 5, 8, 11 and 12 months of age

7.1.3 Other study parameters

Date of birth, gender, duration of pregnancy, birth weight, duration of breast feeding, use of day care, family members and age, smoking habits parents, use of antibiotics last 3 months, current symptoms of cold, ear infections, other disorders.
PCV13 groups: painkiller use around vaccination.

7.2 Randomisation, blinding and treatment allocation

Two randomization lists will be made, one for group 1 and one for group 2.

Prior to starting the trial (group 1) and prior to the 11 month visit (group 2) envelopes which contain a letter indicating allocation to one of the sub groups will be numbered in

random order using a random number generator (www.random.org). The envelopes will be sealed by the principal investigator (who will not be involved in randomization visits) and given to the study team. For group 1 during visit 1, after eligibility has been confirmed and the informed consent form has been signed, the study team member should open the envelope with the lowest number still available. The volunteer will be assigned to the group indicated in the letter. For group 2 the randomization will take place before the 11 months home visits.
In case replacements are warranted due to premature withdrawal of volunteers, after all envelopes have been used, the sponsor will prepare a new set of sealed and randomly numbered envelopes corresponding to the number of required replacements.
For group 1 at the time of randomization of a subject the study team member should take the investigational product vial and vaccinate the subject with the product(s) as indicated. The treatment number should be recorded in the CRF.

The study will not be blinded.

7.3 Study procedures

Invitation and enrolment

Based on live birth data from the CBS (http://www.cbs.nl/nl-NL/menu/home/default.htm) the optimal region for the study is assessed. The region will comprise of parts of Utrecht and Noord-Holland and part of Flevoland and Gelderland for group 2. The RIVM-RCP (who send invites for the National vaccination program) uses the region to make an address list and sends this list to the distributor who sends the invites. Invites containing the information leaflet, return card and envelop and a short recommendation letter from the RCP will be send to all addresses on the list.
The parents send in the return card, to show their interest in the study. In response an email will be send with the extra information (including the informed consent form and the general patient brochure from the CCMO).
A telephone call will be made to the parent, to inquire if they are still interested, explain the study in case of questions and to make an appointment. Their address will be registered for the home visit and to send the extra information also on paper.

Inclusion

Inclusion criteria will be checked during the first home visit, according to the criteria in paragraph 4.2 and 4.3

Randomization, see paragraph 7.2

Questionnaire

The study parameters in the questionnaire are as mentioned in paragraph 7.1.3. The questionnaire is part of the CRF and will be completed by the investigator.

Blood sample

In case of partial or complete failure a second attempt may be performed with consent of the parents. The maximum number of blood collection attempts is 2.
In case of resistance by the child the behaviour code of the Dutch Society of Pediatricians will be followed.

For cellular immunity, an 8 ml blood sample is expected to yield 8-10 x 10^6 PBMC.
6 x 10^6 PBMC are minimally required for culturing and analysis in duplicate wells of memory B cells specific for the selected polysaccharides included in the vaccines and controls. 8 ml blood samples will be collected in 2x 4 ml CPT tubes with sodium citrate (Becton Dickinson, San Diego, California, USA).

IgG antibodies

Blood samples will be transported to the CIB and after centrifugation the serum samples will be stored at -80C. Later, the samples will be tested for all 13 vaccine pneumococcal polysaccharides and DTaP-Hib using the X-map Luminex technology. Only 100 ul of blood is needed for these analyses.

Preparation of PBMCs

Specific B cell frequencies will be measured for five to six serotypes.
In the previous KOKKI study the following serotypes were tested: 6B, 14, 19F and 23F. They are present in PCV7, PCV10 and PCV13.
For the current study a selection of the 13 serotypes will be made. Probably 6B and 19F from PCV7, one-two serotypes from the three serotypes added by PCV10, and two serotypes added by PCV13 (probably 6A and 19A). The final selection will be based on the carrier frequencies just before evaluation.
Fresh Peripheral Blood Mononuclear Cells (PBMCs) will be separated from heparinized blood, within 24 hours after collection, by density gradient gel centrifugation in CPT tubes (Becton Dickinson). Cells are washed using PBS + 5% fetal calf serum (FCS;HyClone). PBMC’s were resuspended in AIM-V medium containing 10% FCS and supplemented with penicillin (100 U/ml), streptomycin (100 µg/ml) and L-glutamine (200nM) (Gibco BRL).

B cell stimulation in vitro (memory B cells)

For the indirect ELISPOT, PBMCs will be resuspended and cultured at a concentration of $2x10^6$ cells/ml in AIM-V culture medium in 24-wells plates. PBMCs will be stimulated polyclonally with 3 µg/ml CpG-C, PTO modified (5'-TCG TCG TCG TTC GAA CGA CGT TGA T-3') (Isogen) in the presence of 10 ng/ml IL-2 (strathmann), 10 ng/ml IL-10 (Calbiochem) and 2 ng/ml of pooled polysaccharides (Stathens Serum Institute) for 5 days at 37°C and 5% CO_2. Cells will be harvested by centrifugation, washed with culture medium and tested in antigen-specific ELISPOT assays.

Plasma B cells

To determine plasma cell frequencies, PBMCs are diluted in culture medium to a concentration of $3x10^6$ cells/ml and used in an ELISPOT to examine the number of plasma cells.

ELISPOT assay

Multiscreen Filtration plates were pre-incubated with 35% ethanol for 1 minute, washed and coated with 100 µl PBS containing either 10 µg/ml goat-anti human IgG (SBA), 25 µg/ml used polysaccharide types or PBS only as a negative control. All plates were incubated at 4°C overnight, washed and blocked for at least 20 minutes with PBS containing 5% FCS. Afterwards 3-fold dilutions of the PBMCs suspension (Direct ELISPOT, plasma B cells) were added to the plates at a starting concentration of at least $3x10^5$ cells/well, or stimulated PBMCs (Indirect ELISPOT, memory B cells) at a starting concentration of at least $0.5x10^5$ cells/well up to $2x10^5$ cells/well in AIM-V culture medium and incubated overnight at 37°C and 5% CO_2. After washing with 0.05% Tween 20/PBS, plates were incubated with alkaline phosphatise (AP)-labeled goat-anti human IgG (1:5000) (SBA) for 2-4 hrs at 37°C. After washes (last wash in PBS), plates were incubated with 50 µl substrate solution (1 mM 5-bromo-4-chloro-3-indolyl phosphate in H_2O; Sigma) for 30-60 minutes. Reaction was stopped by washing and dried. Plaques appearing as blue spots were measured as antigen-secreting cells by using an ELISPOT reader and software (CTL Europe).

7.4 Withdrawal of individual subjects

Subjects can leave the study at any time for any reason if they wish to do so without any consequences. The investigator can decide to withdraw a subject from the study for urgent medical reasons.

7.5 Replacement of individual subjects after withdrawal

For sub groups 1a, 1b, 2a and 2b, 33 subjects are collected per sub group (in order to reach 25 evaluable subjects for cellular immunity. The numbers will also enable sufficient samples for antibody concentrations).

Only in case of dropout subject can be replaced:

- Due to the vaccinations of group 1 it is impossible to replace these subjects after all two months vaccinations are given
- In case of being still feasible, subjects in group 2 can be replaced in case subjects drop out during the first visits.

7.6 Follow-up of subjects withdrawn from treatment

Follow-up is only applicable to group 1. When withdrawing before the end of the vaccination schedule, options need to be discussed to make sure that the child receives the necessary vaccinations. A schedule started with PCV13 should be finished with either PCV13 or PCV7 (not by PCV10). Completion of the schedule can be done by the study team, who can give PCV13. If the parents refuse home visits from the study team the well-baby clinic can complete the schedule with PCV7 (since PCV13 is not part of the RVP and the well-baby clinic can therefore not provide PCV13).

7.7 Premature termination of the study

The sponsor is entitled to terminate the study at any time if new data on the safety or efficacy of the product under study becomes available during the study, making further use of the product undesirable, even in a controlled situation. The METC will be informed about such a decision.

The study can be discontinued:

- If the investigator of that site comes into a situation that impedes the further progress of the study and the investigator cannot be replaced or no other solution can be found.
- In case of repeated unacceptable protocol violations.

In case of premature study termination, options should be discussed to ensure complete vaccination series for the children, see chapter 7.6.

8. SAFETY REPORTING

8.1 Section 10 WMO event

In accordance to section 10, subsection 1, of the WMO, the investigator will inform the subjects and the reviewing accredited METC if anything occurs, on the basis of which it appears that the disadvantages of participation may be significantly greater than was foreseen in the research proposal. The study will be suspended pending further review by the accredited METC, except insofar as suspension would jeopardise the subjects' health. The investigator will take care that all subjects are kept informed.

8.2 Adverse and serious adverse events

Adverse events are defined as any undesirable experience occurring to a subject during the study, whether or not considered related to [the investigational product / the experimental treatment]. All adverse events reported spontaneously by the subject or observed by the investigator or his staff will be recorded.

A serious adverse event is any untoward medical occurrence or effect that at any dose:

- results in death;
- is life threatening (at the time of the event);
- requires hospitalisation or prolongation of existing inpatients' hospitalisation;
- results in persistent or significant disability or incapacity;
- is a congenital anomaly or birth defect;
- is a new event of the trial likely to affect the safety of the subjects, such as an unexpected outcome of an adverse reaction, lack of efficacy of an IMP used for the treatment of a life threatening disease, major safety finding from a newly completed animal study, etc.

All SAEs will be reported through the web portal *ToetsingOnline* to the accredited METC that approved the protocol, within 15 days after the sponsor has first knowledge of the serious adverse reactions.
SAEs that result in death or are life threatening should be reported expedited. The expedited reporting will occur not later than 7 days after the responsible investigator has first knowledge of the adverse reaction. This is for a preliminary report with another 8 days for completion of the report.

SAE's and SUSARs will be reported to the CCMO according to the following:

1 Group 1: Report all SUSARs within 7 days during the entire trial

2 All SAE's related to PCV13 and DTaP-IPV-Hib-HepB vaccination (Group 1) or trial related actions (e.g. venapunction, all groups) will be reported within 7 (resulting in death or is life treatening) or 15 days (all other) during the entire trial
3 Group 1: All SAE's related to invasive pneumococcal disease (IPD) will be reported within 7 (resulting in death or is life treatening) or 15 days (all other) during the entire trial
4 Group 1: All unrelated SAEs resulting in death/persistent serious side effects will be reported within 7 days during the entire trial
5 All other unrelated SAEs will be reported semiannual
   a. Group 2: Registration will taken place during one week after blood collection
   b. Group 1:
      i. Registration will take place in the period of trial entry until one month after the primary series (in practice until ~5 months of age)
      ii. During the period of pre-booster blood collection until one month after the booster vaccination
   c. The report will contain the following: subject number, vaccination date, SAE start and stop date, diagnosis, severity, relation to vaccination or study procedure

8.2.1 Suspected unexpected serious adverse reactions (SUSAR)

Adverse reactions are all untoward and unintended responses to an investigational product related to any dose administered.

Unexpected adverse reactions are adverse reactions, of which the nature, or severity, is not consistent with the applicable product information (e.g. Investigator's Brochure for an unapproved IMP or Summary of Product Characteristics (SPC) for an authorised medicinal product).

The sponsor will report expedited the following SUSARs through the web portal *ToetsingOnline* to the METC:

- SUSARs that have arisen in the clinical trial that was assessed by the METC;
- SUSARs that have arisen in other clinical trials of the same sponsor and with the same medicinal product, and that could have consequences for the safety of the subjects involved in the clinical trial that was assessed by the METC.

The remaining SUSARs are recorded in an overview list (line-listing) that will be submitted once every half year to the METC. This line-listing provides an overview

of all SUSARs from the study medicine, accompanied by a brief report highlighting the main points of concern.
The expedited reporting of SUSARs through the web portal ToetsingOnline is sufficient as notification to the competent authority.

The sponsor will report expedited all SUSARs to the competent authorities in other Member States, according to the requirements of the Member States.

The expedited reporting will occur not later than 15 days after the sponsor has first knowledge of the adverse reactions. For fatal or life threatening cases the term will be maximal 7 days for a preliminary report with another 8 days for completion of the report.

8.2.2 Annual safety report

Not applicable

8.3 Follow-up of adverse events

All adverse events will be followed until they have abated, or until a stable situation has been reached. Depending on the event, follow up may require additional tests or medical procedures as indicated, and/or referral to the general physician or a medical specialist.

8.4 Data Safety Monitoring Board (DSMB)

Not applicable.

9. STATISTICAL ANALYSIS

9.1 Descriptive statistics

Baseline comparability of the groups will be shown by descriptive statistics and frequency tables (e.g. with information as gender, age, day care attendance etc).

Little is known about cellular immune responses against *Streptococcus pneumonia* quantitative endpoints of this study.

Frequencies of pneumococcal specific B cell populations (number of cells per 10^6 PBMC) and antibody concentrations (U/ml) will be calculated per patient.

B cell frequencies will be shown in medians and will be compared using non-parametric Mann-Whitney test. Results of antibody levels are expressed in geometric mean concentration (GMC) with 95% confidence interval (95% CI). Statistical differences in IgG GMC values will be assessed by log transformed unpaired t test. Correlations between IgG levels and circulating B cells will be assessed by Spearman correlation. All reported p-values are 2-sided, p-values smaller then 0.05 are considered significant. Analyses will be performed with SPSS 15.0 and PRISM4.

Other more qualitative endpoints, such as avidity and opsonophagosytoses, will be described as such.

9.2 Univariate analysis

For group 2 of the currents study and the PCV13 group of the PIM study (vaccination at 2, 3, 4 and 11 months of age), differences in serotype specific antibody concentrations between different schedules will be analyzed. Primary, the antibody concentrations against pneumococcal polysaccharides for each serotype at 12 months in the different study arms will be calculated. GMCs and the degree of protection (the proportion with concentration > 0.35 µg/ml) will be determined.

Secondary, the pneumococcal antibody concentrations at 5, 8, immediately before and 7-9 days after the 11-months booster and at 12 months of age will be calculated. For these endpoints, GMCs and the degree of protection (the proportion with concentration > 0.35 µg/ml) will be determined. The antibody concentrations of the longitudinal samples of each child will be used to assess the kinetics.

Also the DTaP-Hib antibody concentrations at 5, 8, immediately before and 7-9 days after the 11-months booster and at 12 months of age will be calculated.

A chi-square test will be used to determine differences in proportions. A T-test or a distribution free variable will be used to test differences in mean or median.

9.3 Multivariate analysis

Modifying factors, like family structure etc. will be analyzed for exploratory reasons only, in a multivariate regression analyses with as primary outcome measure antibody concentrations against the 13 serotypes *S. pneumoniae*.

10. ETHICAL CONSIDERATIONS

10.1 Regulation statement

This clinical study will be performed conform the current rules for GCP, as described by the Committee for Proprietary Medical Products (CPMP) of the European Union and the International Committee on Harmonisation (ICH) in "Note for Guidance on Good Clinical Practice, document CPMP/ICH/135/95", effective since January 17th 1997, Clinical Trial Directive 2001/20/EC, The World Medical Declaration of Helsinki and its amendments effective since 1964 (last updated 9 October 2004) and according to national legal and regulatory requirements.

10.2 Recruitment and consent

See 7.3 for a detailed overview of events. Before the first study appointment parents/legal representatives will receive an information leaflet with a response card (Annex 1). Following a positive response more detailed patient information with informed consent forms will be sent (Annex 2). The parent(s)/legal representative(s) will then be contacted by phone to discuss the study, answer questions and to make an appointment for the first home visit. The parent(s)/legal representative(s) will have at least one week between the phone call and the home visit where they sign the informed consent.
Study procedures will only take place after both parents/legal representative(s) have signed the informed consent (one parent in case of an orphan, or single-parent family).

10.3 Objection by minors or incapacitated subjects (if applicable)

Parents are at all times allowed to withdraw the informed consent. The parent and the investigator can at all times decide to end the participation of the child if the child shows resistance to the study procedure. The code of conduct will be followed which is published by the Nederlandse Vereniging voor Kindergeneeskunde 'Gedragscode bij verzet van minderjarigen die deelnemen aan medisch-wetenschappelijk onderzoek' .
The right of the parents to withdraw informed consent at all times is put down in the informed consent letter.

10.4 Benefits and risks assessment, group relatedness

Blood collection: one blood collection of 8 ml (2x4 ml tubes). The burden and risk is considered low.
The children might find the needle scary and it might be painful (only for a few seconds). A local anaesthetic (Emla® crème, Astra Zeneca) may be used to minimize pain. Blood

collection could result in a small bruise at the location of injection, which will disappear within a few days.
Group 1; one heel/finger stick sampling, group 2: 3-4: heel/finger sticks samplings. The burden and risk is considered low.

For group 2 (PCV10 group), the children themselves have no direct benefit in participating in this trial. The trial is aimed to study the (cellular) immune response after 4 PCV10 or PCV13 vaccinations. These children, who have followed the Dutch NIP, are the only possible children that can participate in the trial. Visits will take 10-30 minutes each (depending on the type of blood collection and whether a questionnaire is taken).

Children in group 1 will receive PCV13 vaccinations. The side effects of these vaccinations are expected to be equal to the side effects of PCV10 (which the children would have received as part of the NIP). They will however receive these vaccinations at home to reduce the study burden. These children will benefit from the added protection of the three extra serotypes which are not present in the PCV10 vaccination. These children are the only possible study group, since they are eligible for the Dutch NIP. Visits will take maximum 30 minutes each.

10.5 Compensation for injury

According to a Ministerial Order, RIVM is excluded from compulsory insurance for clinical research as determined by the Dutch law on Medical Investigations (WMO, section 7, paragraph 6). Participants can recover the loss from RIVM. Any claims will be settled according to the same terms that an insurance company uses.

Normal participants insurance provides cover for damage to research subjects through injury or death caused by the study.

1. € 450.000,-- (i.e. four hundred and fifty thousand Euro) for death or injury for each subject who participates in the Research;
2. € 3.500.000,-- (i.e. three million five hundred thousand Euro) for death or injury for all subjects who participate in the Research;
3. € 5.000.000,-- (i.e. five million Euro) for the total damage incurred by the organisation for all damage disclosed by scientific research for the Sponsor as ‘verrichter’ in the meaning of said Act in each year of insurance coverage.

The insurance applies to the damage that becomes apparent during the study or within 4 years after the end of the study.

10.6 Incentives

All children will receive one or two small presents during some visits. The maximum amount spend on presents will be 15 euro per child.

11. ADMINISTRATIVE ASPECTS AND PUBLICATION

11.1 Handling and storage of data and documents

All children participating in the study will receive a unique subject number, a difference is made between PCV13 and PCV10 groups to allow for prioritizing the serotypes tested in case of low amounts of blood:

- Group 1: COP13_001 - COP13_066
- Group 25: COP10_101 - COP10_166

Al trial data is recorded using this subject number and is saved for 15 years according to legal requirements.
All recorded data is treated confidential such that data in reports or other publications of the trial can never be traced back to the child or family. Recorded data can only be accessed by competent and qualified research employees, by members of the CCMO or by representatives of the sponsor and the competent authorities.

11.2 Amendments

Amendments are changes made to the research after a favourable opinion by the accredited METC has been given. All amendments will be notified to the METC that gave a favourable opinion.

A ‘substantial amendment’ is defined as an amendment to the terms of the METC application, or to the protocol or any other supporting documentation, that is likely to affect to a significant degree:

- the safety or physical or mental integrity of the subjects of the trial;
- the scientific value of the trial;
- the conduct or management of the trial; or
- the quality or safety of any intervention used in the trial.

All substantial amendments will be notified to the METC and to the competent authority.

Non-substantial amendments will not be notified to the accredited METC and the competent authority, but will be recorded and filed by the sponsor.

11.3 Annual progress report

The sponsor/investigator will submit a summary of the progress of the trial to the accredited METC once a year. Information will be provided on the date of inclusion of the

first subject, numbers of subjects included and numbers of subjects that have completed the trial, serious adverse events/ serious adverse reactions, other problems, and amendments.

11.4 End of study report

The sponsor will notify the accredited METC and the competent authority of the end of the study within a period of 90 days. The end of the study is defined as the last patient's last visit.

In case the study is ended prematurely, the sponsor will notify the accredited METC and the competent authority within 15 days, including the reasons for the premature termination.

Within one year after the end of the study, the investigator/sponsor will submit a final study report with the results of the study, including any publications/abstracts of the study, to the accredited METC and the Competent Authority.

11.5 Public disclosure and publication policy

The study results will be reported in an internal report and submitted for publication in peer-reviewed journals. Publications will be drafted by the sponsor investigators.

12. REFERENCES

1. Oosterhuis-Kafeja F, Beutels P, Van Damme P. Immunogenicity, efficacy, safety and effectiveness of pneumococcal conjugate vaccines (1998-2006). Vaccine. 2007 Mar 8;25(12):2194-212.
2. Bogaert D, van Belkum A, Sluijter M, Luijendijk A, de Groot R, Rumke HC, et al. Colonisation by Streptococcus pneumoniae and Staphylococcus aureus in healthy children. Lancet. 2004 Jun 5;363(9424):1871-2.
3. Syrjanen RK, Kilpi TM, Kaijalainen TH, Herva EE, Takala AK. Nasopharyngeal carriage of Streptococcus pneumoniae in Finnish children younger than 2 years old. J Infect Dis. 2001 Aug 15;184(4):451-9.
4. WHO. Recommendations to assure the quality, safety and efficacy of pneumococcal conjugate vaccines. Expert Committee on Biological Standardization. 2009.
5. Kieninger DM, Kueper K, Steul K, Juergens C, Ahlers N, Baker S, et al. Safety, tolerability, and immunologic noninferiority of a 13-valent pneumococcal conjugate vaccine compared to a 7-valent pneumococcal conjugate vaccine given with routine pediatric vaccinations in Germany. Vaccine. 2010 Jun 7;28(25):4192-203.
6. Prymula R, Chlibek R, Splino M, Kaliskova E, Kohl I, Lommel P, et al. Safety of the 11-valent pneumococcal vaccine conjugated to non-typeable Haemophilus influenzae-derived protein D in the first 2 years of life and immunogenicity of the co-administered hexavalent diphtheria, tetanus, acellular pertussis, hepatitis B, inactivated polio virus, Haemophilus influenzae type b and control hepatitis A vaccines. Vaccine. 2008 Aug 18;26(35):4563-70.
7. Gourley TS, Wherry EJ, Masopust D, Ahmed R. Generation and maintenance of immunological memory. Semin Immunol. 2004 Oct;16(5):323-33.
8. Goldblatt D, Southern J, Ashton L, Richmond P, Burbidge P, Tasevska J, et al. Immunogenicity and boosting after a reduced number of doses of a pneumococcal conjugate vaccine in infants and toddlers. Pediatr Infect Dis J. 2006 Apr;25(4):312-9.
9. Vesikari T, Wysocki J, Chevallier B, Karvonen A, Czajka H, Arsene JP, et al. Immunogenicity of the 10-valent pneumococcal non-typeable Haemophilus influenzae protein D conjugate vaccine (PHiD-CV) compared to the licensed 7vCRM vaccine. Pediatr Infect Dis J. 2009 Apr;28(4 Suppl):S66-76
10 .Perneger TV. What's wrong with Bonferroni adjustments. BMJ. 1998 Apr18;316(7139): 1236-8.
