# Supplementary material for: Direct Comparison of Immunogenicity Induced by 10- or 13-Valent Pneumococcal Conjugate Vaccine around the 11-Month Booster in Dutch Infants
Source: PLoS One. 2015 Dec 10;10(12):e0144739. doi: 10.1371/journal.pone.0144739 (PMC4690595; doi:10.1371/journal.pone.0144739)
Supplement: S1 Table — (PDF) [file pone.0144739.s003.pdf]

S1 Table Geometric mean concentrations (GMC) with 95% CI of the antibody concentrations against 13 pneumococcal serotypes for the PCV10 group and the PCV13 group with p-values for differences between the groups and crude and adjusted GMC ratios with 95% CI comparing PCV13 with PCV10

	Pre-booster					One week post-booster					One month post-booster				
	PCV13 (N=31)	PCV10 (N=57)				PCV13 (N=29)	PCV10 (N=28)				PCV13 (N=65)	PCV10 (N=66)			
Sero-type	GMC (95% CI)	GMC (95% CI)	p-value	Crude GMC ratio (95% CI)	Adjusted GMC ratio* (95% CI)	GMC (95% CI)	GMC (95% CI)	p-value	Crude GMC ratio (95% CI)	Adjusted GMC ratio** (95% CI)	GMC (95% CI)	GMC (95% CI)	p-value	Crude GMC ratio (95% CI)	Adjusted GMC ratio** (95% CI)
1	0.67	1.05	0.031	0.64	0.70	16.98	22.75	0.237	0.75	0.69	11.67	12.20	0.787	0.96	0.98
	(0.53-0.86)	(0.80-1.36)		(0.44-0.95)	(0.39-1.27)	(11.86-24.30)	(16.01-32.34)		(0.46-1.21)	(0.37-1.29)	(9.33-14.60)	(9.60-15.51)		(0.69-1.32)	(0.63-1.52)
4	0.24	0.30	0.123	0.79	0.73	4.50	4.97	0.641	0.90	0.90	3.25	3.30	0.912	0.98	0.90
	(0.18-0.32)	(0.26-0.36)		(0.58-1.06)	(0.46-1.16)	(3.13-6.46)	(3.91-6.34)		(0.59-1.38)	(0.51-1.61)	(2.57-4.10)	(2.69-4.05)		(0.73-1.33)	(0.59-1.36)
5	0.93	0.87	0.677	1.07	1.31	14.00	10.77	0.228	1.30	1.30	11.38	6.67	<0.001	1.71	1.72
	(0.71-1.22)	(0.72-1.06)		(0.78-1.48)	(0.82-2.10)	(9.89-19.81)	(8.24-14.08)		(0.85-1.98)	(0.75-2.26)	(9.38-13.81)	(5.52-8.06)		(1.31-2.22)	(1.19-2.47)
6B	0.41	1.07	<0.001	0.38	0.30	13.99	12.75	0.681	1.10	1.21	9.98	6.97	0.013	1.43	1.55
	(0.29-0.56)	(0.87-1.33)		(0.26-0.54)	(0.18-0.52)	(9.41-20.78)	(10.21-15.92)		(0.71-1.70)	(0.65-2.23)	(8.00-12.47)	(5.83-8.34)		(1.08-1.89)	(1.06-2.27)
7F	2.13	2.29	0.645	0.93	0.91	16.66	24.64	0.109	0.68	0.81	14.99	15.89	0.685	0.94	0.99
	(1.73-2.63)	(1.88-2.78)		(0.69-1.26)	(0.58-1.42)	(10.97-25.30)	(19.15-31.69)		(0.42-1.08)	(0.43-1.52)	(11.97-18.75)	(13.28-19.00)		(0.71-1.25)	(0.67-1.46)
9V	0.52	1.17	<0.001	0.45	0.51	16.18	15.45	0.797	1.05	1.19	10.90	8.64	0.045	1.26	1.45
	(0.41-0.66)	(0.98-1.39)		(0.33-0.59)	(0.33-0.78)	(12.40-21.11)	(12.00-19.88)		(0.74-1.49)	(0.74-1.92)	(9.31-12.75)	(7.32-10.20)		(1.01-1.58)	(1.07-1.97)
14	1.75	1.09	0.059	1.61	1.62	15.09	9.86	0.059	1.53	1.41	13.92	7.21	<0.001	1.93	1.75
	(1.19-2.60)	(0.80-1.47)		(0.99-2.63)	(0.80-3.29)	(11.51-19.79)	(6.85-14.21)		(0.99-2.36)	(0.79-2.53)	(11.35-17.07)	(5.72-9.10)		(1.42-2.62)	(1.17-2.64)
18C	0.66	1.04	0.004	0.64	0.52	15.23	16.27	0.728	0.94	1.17	9.81	12.50	0.067	0.78	0.84
	(0.54-0.82)	(0.85-1.26)		(0.47-0.86)	(0.34-0.80)	(11.26-20.60)	(12.81-20.65)		(0.65-1.35)	(0.71-1.92)	(8.02-12.00)	(10.56-14.79)		(0.61-1.02)	(0.59-1.19)
19F	1.25	0.52	0.002	2.41	1.14	44.98	2.78	<0.001	16.19	17.64	24.37	2.09	<0.001	11.65	8.39
	(0.75-2.11)	(0.38-0.71)		(1.39-4.17)	(0.52-2.54)	(30.40-66.55)	(1.51-5.11)		(8.15-32.16)	(7.17-43.37)	(18.54-32.03)	(1.40-3.12)		(7.22-18.78)	(4.42-15.93)
23F	0.44	0.93	0.013	0.47	0.35	19.44	14.32	0.225	1.36	1.32	12.35	7.00	0.002	1.77	1.92
	(0.26-0.74)	(0.66-1.30)		(0.27-0.84)	(0.15-0.82)	(13.04-28.97)	(10.45-19.62)		(0.83-2.21)	(0.75-2.35)	(9.52-16.03)	(5.52-8.87)		(1.25-2.49)	(1.21-3.05)
3	0.47	0.25	0.005	1.88	1.53	4.80	0.16	<0.001	29.19	33.82	3.86	0.44	<0.001	8.83	9.97
	(0.36-0.60)	(0.18-0.33)		(1.22-2.88)	(0.80-2.91)	(3.34-6.91)	(0.14-0.20)		(19.69-43.28)	(19.49-58.67)	(3.19-4.67)	(0.33-0.59)		(6.27-12.45)	(6.24-15.94)
6A	0.74	0.12	<0.001	6.31	3.41	37.86	1.55	<0.001	24.45	21.14	22.36	0.75	<0.001	29.62	26.88
	(0.51-1.08)	(0.08-0.18)		(3.46-11.49)	(1.42-8.20)	(27.25-52.61)	(0.89-2.68)		(13.33-44.83)	(9.11-49.05)	(18.22-27.44)	(0.51-1.13)		(19.03-46.09)	(14.74-49.01)
19A	0.63	0.28	0.002	2.22	1.16	15.92	0.89	<0.001	17.94	18.98	8.90	0.79	<0.001	11.25	8.43
	(0.39-1.01)	(0.21-0.37)		(1.35-3.64)	(0.56-2.40)	(10.33-24.54)	(0.50-1.59)		(9.00-35.78)	(7.72-46.66)	(6.76-11.72)	(0.55-1.13)		(7.22-17.55)	(4.65-15.29)

*Adjusted for age in days at 1st, 2nd, 3rd vaccination and age at 11-month blood sampling

**Adjusted for age in days at 1st, 2nd, 3rd and 4th vaccination
