# Supplementary material for: Direct Comparison of Immunogenicity Induced by 10- or 13-Valent Pneumococcal Conjugate Vaccine around the 11-Month Booster in Dutch Infants
Source: PLoS One. 2015 Dec 10;10(12):e0144739. doi: 10.1371/journal.pone.0144739 (PMC4690595; doi:10.1371/journal.pone.0144739)
Supplement: S2 Table — (PDF) [file pone.0144739.s004.pdf]

S2 table Mean avidity index with 95% CI for 13 pneumococcal serotypes for the PCV10 group and the PCV13 group with p-values for differences between the groups and crude and adjusted mean difference with 95% CI comparing PCV13 with PCV10

	Pre-booster					One week post-booster					One month post-booster				
**Serotype**	PCV13 (N=29)	PCV10 (N=55)		Crude mean difference	Adjusted mean difference	PCV13 (N=29)	PCV10 (N=26)		Crude mean difference	Adjusted mean difference	PCV13 (N=64)	PCV10 (N=66)		Crude mean difference	Adjusted mean difference
	Mean	Mean	p-value	(95% CI)	(95% CI)	Mean	Mean	p-value	(95% CI)	(95% CI)	Mean	Mean	p-value	(95% CI)	(95% CI)
**1**	59	62	0.262	-3.0	-1.7	51	63	0.008	-12.1	-13.4	54	61	0.018	-6.1	-5.8
	(55-64)	(59-65)		(-8.1-2.2)	(-9.5-6.1)	(46-57)	(56-71)		(-20.6 - -3.5)	(-25.5 - -1.3)	(51-58)	(57-64)		(-11.3--0.9)	(-12.9-1.3)
**4**	66	70	0.121	-4.5	-4.6	74	78	0.281	-4.6	-9.5	75	73	0.576	1.7	-1.3
	(61-70)	(67-74)		(-10.2-1.1)	(-13.2-4.0)	(68-79)	(71-85)		(-12.9-3.7)	(-20.5-1.6)	(71-78)	(69-77)		(-3.4-6.8)	(-8.3-5.7)
**5**	72	80	0.002	-7.5	-6.1	70	84	<0.001	-14.9	-14.9	74	82	0.001	-7.9	-6.9
	(67-77)	(77-82)		(-12.0 - -3.0)	(-12.9-0.8)	(64-75)	(80-89)		(-22.1 - -7.8)	(-24.8 - -5.1)	(70-78)	(79-85)		(-12.3--3.5)	(-13.0 - -0.9)
**6B**	63	59	0.261	3.9	0.8	63	63	0.950	-0.3	-8.2	65	59	0.016	6.6	5.4
	(58-68)	(55-63)		(-2.9-10.7)	(-9.5-11.1)	(57-69)	(57-70)		(-8.7-8.1)	(-19.4-3.0)	(61-68)	(55-62)		(1.4-11.7)	(-1.7-12.4)
**7F**	80	84	0.068	-3.5	-3.5	77	86	0.003	-9.0	-14.0	78	85	<0.001	-7.2	-6.8
	(77-83)	(81-86)		(-7.2-0.2)	(-9.1-2.2)	(73-81)	(81-90)		(-14.6 - -3.3)	(-21.4 - -6.5)	(75-81)	(83-88)		(-11.0--3.4)	(-12.0 - -1.5)
**9V**	78	79	0.627	-1.1	0.5	81	82	0.834	-0.7	-4.5	82	79	0.196	2.9	4.2
	(74-82)	(76-82)		(-5.8-3.5)	(-6.7-7.6)	(78-85)	(76-87)		(-6.8-5.5)	(-12.4-3.5)	(80-85)	(76-83)		(-1.2-6.9)	(-1.4-9.8)
**14**	74	81	0.013	-6.7	-8.7	74	86	0.005	-11.7	-11.9	75	83	0.001	-8.4	-11.7
	(69-79)	(78-84)		(-11.9 - -1.5)	(-16.4 - -1.0)	(67-81)	(81-90)		(-19.5 - -3.9)	(-22.9 - -0.8)	(71-78)	(80-86)		(-13.1 - -3.7)	(-18.0 - -5.5)
**18C**	65	64	0.805	0.7	-4.2	73	77	0.201	-4.3	-8.8	70	76	0.020	-5.4	-7.6
	(61-68)	(61-68)		(-4.6-5.9)	(-12.2-3.7)	(69-78)	(72-82)		(-10.8-2.2)	(-17.6-0.0)	(67-74)	(73-79)		(-10.0 - -0.9)	(-13.8 - -1.4)
**19F**	61	48	0.003	12.2	13.4	68	46	<0.001	22.1	12.7	66	48	0.001	17.8	17.3
	(56-65)	(43-54)		(4.4-20.0)	(1.4-25.4)	(62-74)	(38-54)		(12.4-31.8)	(-0.7-26.0)	(62-70)	(43-52)		(11.5-24.1)	(8.5-26.1)
**23F**	65	67	0.385	-2.7	0.3	69	73	0.393	-3.7	-4.6	75	71	0.385	4.0	5.6
	(59-70)	(64-71)		(-8.8-3.4)	(-8.6-9.2)	(63-75)	(66-79)		(-12.3-4.8)	(-15.6-6.4)	(72-79)	(68-75)		(-0.8-8.9)	(-1.1-12.2)
**3**	60	nd*				48	nd				47	nd			
	(56-63)	nd				(41-55)	nd				(43-52)	nd			
**6A**	58	nd				62	nd				64	nd			
	(53-63)	nd				(56-69)	nd				(59-68)	nd			
**19A**	59	nd				69	nd				66	nd			
	(54-65)	nd				(63-75)	nd				(62-70)	nd			

*Avidity was not determined in the PCV10 group for serotype 3, 6A and 19A
