# Supplementary material for: Direct Comparison of Immunogenicity Induced by 10- or 13-Valent Pneumococcal Conjugate Vaccine around the 11-Month Booster in Dutch Infants
Source: PLoS One. 2015 Dec 10;10(12):e0144739. doi: 10.1371/journal.pone.0144739 (PMC4690595; doi:10.1371/journal.pone.0144739)
Supplement: S3 Table — (PDF) [file pone.0144739.s005.pdf]

S3 Table Geometric mean titers (GMT) with 95% CI of opsonophagocytosis for 13 pneumococcal serotypes for the PCV10 group and the PCV13 group with p-values for differences between the groups and crude and adjusted GMT ratios with 95% CI comparing PCV13 with PCV10

	**Pre-booster**					**One week post-booster**				
	**PCV13 (N=25-31)**	**PCV10 (N=22-27)**				**PCV13 (N=28)**	**PCV10 (N=27)**			
Serotype	GMT (95% CI)	GMT (95% CI)	p-value	Crude GMC ratio (95% CI)	Adjusted GMC ratio* (95% CI)	GMT (95% CI)	GMT (95% CI)	p-value	Crude GMC ratio (95% CI)	Adjusted GMC ratio** (95% CI)
1	4.1 (3.8-4.5)	4.9 (3.6-6.8)	0.254	0.84 (0.62-1.13)	1.14 (0.76-1.73)	267 (157-455)	350 (236-520)	0.408	0.76 (0.40-1.44)	0.54 (0.23-1.26)
4	14.0 (6.5-30.0)	27.8 (16.2-47.7)	0.132	0.50 (0.21-1.21)	0.51 (0.13-2.01)	3661 (2465-5439)	3427 (2605-4507)	0.780	1.07 (0.67-1.70)	1.18 (0.62-2.25)
5	8.0 (5.7-11.4)	8.3 (5.8-11.9)	0.895	0.97 (0.60-1.57)	1.45 (0.69-3.06)	556 (380-815)	347 (250-480)	0.060	1.60 (0.99-2.59)	1.64 (0.87-3.08)
6B	18.6 (10.0-34.8)	112.0 (54.7-229.4)	<0.001	0.17 (0.07-0.41)	0.16 (0.04-0.67)	5173 (3633-7367)	4536 (3369-6107)	0.563	1.14 (0.73-1.78)	1.27 (0.68-2.37)
7F	471.1 (360.1-616.2)	481.8 (329.2-705.2)	0.920	0.98 (0.63-1.51)	1.54 (0.81-2.91)	7850 (5648-10910)	5491 (4320-6979)	0.079	1.43 (0.97-2.11)	1.29 (0.75-2.21)
9V	38.0 (16.9-85.6)	22.8 (11.7-44.5)	0.332	1.67 (0.60-4.64)	2.39 (0.46-12.26)	6274 (3671-10725)	3039 (2062-4479)	0.030	2.06 (1.09-3.90)	1.83 (0.75-4.43)
14	387.4 (222.6-674.1)	280.2 (135.1-581.0)	0.464	1.38 (0.58-3.27)	2.88 (0.78-10.65)	6461 (4504-9271)	5442 (3650-8113)	0.515	1.19 (0.71-1.98)	1.38 (0.67-2.87)
18C	66.3 (30.6-143.6)	109.3 (57.3-208.4)	0.323	0.61 (0.23-1.62)	0.74 (0.16-3.43)	7328 (5349-10039)	4839 (3584-6533)	0.056	1.51 (1.00-2.29)	1.77 (0.98-3.20)
19F	25.8 (11.8-56.1)	136.1 (72.7-254.6)	0.001	0.19 (0.07-0.50)	0.21 (0.05-0.85)	4649 (3196-6763)	3035 (1421-6480)	0.300	1.53 (0.69-3.40)	1.47 (0.47-4.62)
23F	48.5 (19.1-123.3)	108.8 (57.6-205.8)	0.157	0.45 (0.15-1.34)	0.40 (0.08-2.09)	9002 (6210-13048)	4540 (3615-5701)	0.002	1.98 (1.30-3.02)	2.52 (1.39-4.56)
3	6.5 (4.8-8.6)	9.5 (6.2-14.5)	0.117	0.68 (0.42-1.09)	0.75 (0.35-1.61)	154 (105-227)	12 (7-21)	<0.001	12.76 (6.85-23.77)	14.95 (6.28-35.56)
6A	175.2 (93.1-329.7)	61.2 (21.0-178.2)	0.073	2.87 (0.93-8.83)	1.69 (0.27-10.71)	18094 (13293-24629)	2888 (1812-4602)	<0.001	6.27 (3.69-10.64)	6.46 (3.02-13.82)
19A	17.8 (8.0-39.6)	10.0 (4.9-20.4)	0.280	1.78 (0.63-5.04)	0.84 (0.18-3.98)	4516 (3031-6729)	285 (139-585)	<0.001	15.85 (7.29-34.48)	34.00 (12.26-94.32)

*Adjusted for age in days at 1st, 2nd, 3rd vaccination and age at 11-month blood sampling

**Adjusted for age in days at 1st, 2nd, 3rd and 4th vaccination
