# Supplementary material for: Direct Comparison of Immunogenicity Induced by 10- or 13-Valent Pneumococcal Conjugate Vaccine around the 11-Month Booster in Dutch Infants
Source: PLoS One. 2015 Dec 10;10(12):e0144739. doi: 10.1371/journal.pone.0144739 (PMC4690595; doi:10.1371/journal.pone.0144739)
Supplement: S4 Table — (PDF) [file pone.0144739.s006.pdf]

S4 table Geometric mean concentrations (GMC) with 95% CI of the antibody concentrations against diphtheria, tetanus, pertussis and Hib antigens for the PCV10 group and the PCV13 group with p-values for differences between the groups and crude and adjusted GMC ratios with 95% CI comparing PCV13 with PCV10

**Antigen**	**Pre-booster** **PCV13 (N=31)** GMC (95% CI)	**PCV10 (N=56)** GMC (95% CI)	p-value	Crude GMC ratio (95% CI)	Adjusted GMC ratio* (95% CI)	**One month post-booster** **PCV13 (N=65)** GMC (95% CI)	**PCV10 (N=66)** GMC (95% CI)	p-value	Crude GMC ratio (95% CI)	Adjusted GMC ratio*** (95% CI)
Diphtheria	0.11	0.12	0.577	0.91	1.01	1.73	1.80	0.672	0.96	1.06
	(0.08-0.15)	(0.10-0.14)		(0.65-1.27)	(0.62-1.66)	(1.49-2.00)	(1.56-2.08)		(0.78-1.17)	(0.81-1.39)
Pertussis FHA	22.63	27.39	0.350	0.83	1.43	153.41	180.46	0.142	0.85	0.98
	(16.02-31.95)	(21.58-34.77)		(0.55-1.23)	(0.84-2.44)	(131.86-178.47)	(153.96-211.53)		(0.69-1.05)	(0.73-1.32)
Pertussis PRN	16.72	20.09	0.386	0.83	0.98	301.86	303.01	0.979	1.00	1.19
	(11.63-24.02)	(15.72-25.68)		(0.55-1.26)	(0.55-1.74)	(243.47-374.25)	(247.58-370.84)		(0.75-1.33)	(0.80-1.77)
Pertussis PT	17.05	16.17	0.730	1.05	0.94	142.04	119.38	0.149	1.19	0.99
	(12.97-22.42)	(13.60-19.23)		(0.78-1.42)	(0.61-1.45)	(118.13-170.80)	(102.50-139.03)		(0.94-1.50)	(0.72-1.36)
Tetanus	0.30	0.61	<0.001	0.49	0.51	2.98	5.55	<0.001	0.54	0.59
	(0.22-0.42)	(0.53-0.70)		(0.36-0.67)	(0.33-0.80)	(2.51-3.54)	(4.76-6.466)		(0.43-0.67)	(0.43-0.80)
Hib**	0.04	0.28	<0.001	0.14	0.12	10.28	17.00	0.053	0.60	0.46
	(0.02-0.07)	(0.20-0.40)		(0.08-0.26)	(0.05-0.29)	(6.81-15.53)	(12.48-23.16)		(0.36-1.00)	(0.23-0.91)

*Adjusted for age in days at 1st, 2nd, 3rd vaccination and age at 11-month blood sampling

**N=29 for PCV13 (pre-booster), N=65 for PCV10 (one month post-booster)

***Adjusted for age in days at 1st, 2nd, 3rd and 4th vaccination
