# Supplementary material for: Direct Comparison of Immunogenicity Induced by 10- or 13-Valent Pneumococcal Conjugate Vaccine around the 11-Month Booster in Dutch Infants
Source: PLoS One. 2015 Dec 10;10(12):e0144739. doi: 10.1371/journal.pone.0144739 (PMC4690595; doi:10.1371/journal.pone.0144739)
Supplement: S5 Table — (PDF) [file pone.0144739.s007.pdf]

S5 Table. Spearman correlations one week post-booster between IgG, OPA titer and avidity for the PCV10 group and the PCV13 group

	IgG and OPA		Avidity and OPA		IgG and avidity	
**Serotype**	**PCV13**	**PCV10**	**PCV13**	**PCV10**	**PCV13**	**PCV10**
1	0.89	0.87	0.25	0.73	0.26	0.61
4	0.71	0.38	0.31	-0.31	0.29	0.09
5	0.71	0.88	0.36	0.35	0.39	0.43
6B	0.82	0.54	-0.02	-0.12	0.22	0.24
7F	0.34	0.33	0.17	-0.18	0.32	-0.24
9V	0.26	0.33	-0.02	-0.15	-0.01	0.02
14	0.17	0.61	0.05	-0.27	0.03	-0.35
18C	0.66	0.41	0.4	-0.07	0.32	0.22
19F	0.49	0.39	0.35	-0.04	0.41	-0.02
23F	0.52	0.03	-0.08	-0.03	0.28	0.33
3	0.66	0.34	0.07	na	0.23	na
6A	0.6	0.43	0.06	na	0.36	na
19A	0.75	0.57	0.49	na	0.4	na
