# Supplementary material for: Direct Comparison of Immunogenicity Induced by 10- or 13-Valent Pneumococcal Conjugate Vaccine around the 11-Month Booster in Dutch Infants
Source: PLoS One. 2015 Dec 10;10(12):e0144739. doi: 10.1371/journal.pone.0144739 (PMC4690595; doi:10.1371/journal.pone.0144739)
Supplement: S1 Fig — (PDF) [file pone.0144739.s008.pdf]

S1 Figure. Spearman correlations between IgG, OPA titer and avidity for the PCV10-group and the PCV13-group

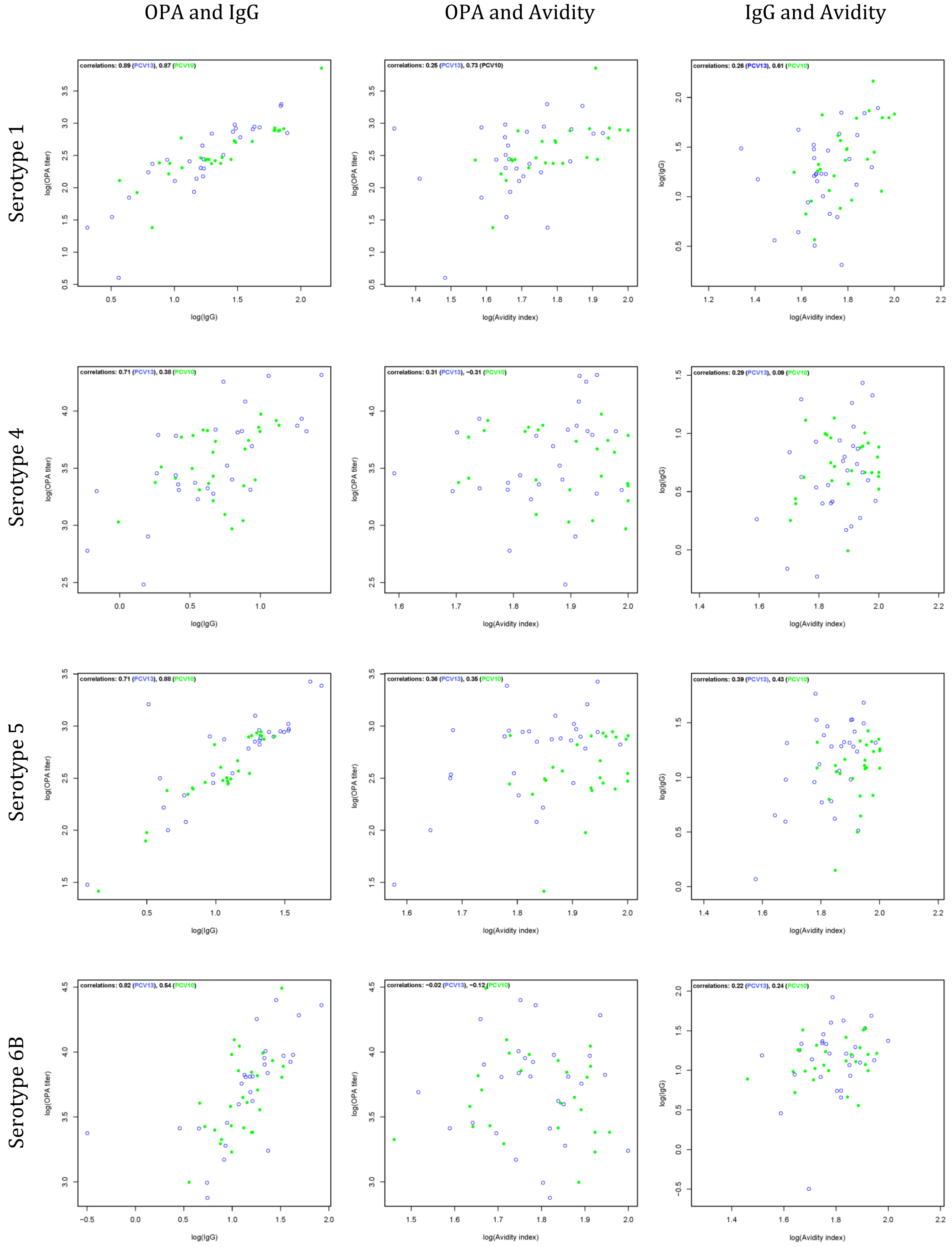

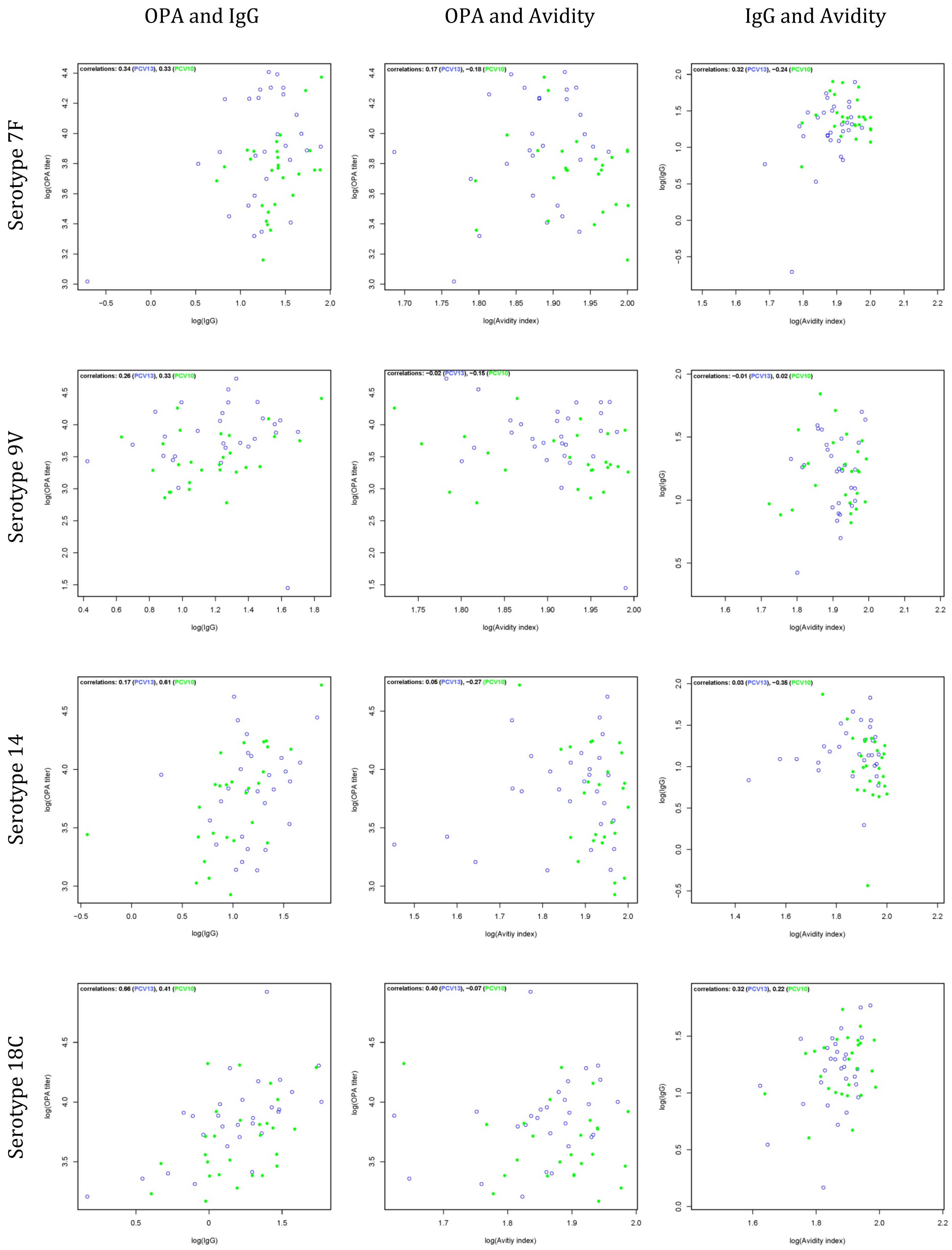
OPA and IgG
OPA and Avidity
IgG and Avidity
Serotype 7F
correlations: 0.34 (PCV13), 0.33 (PCV10)
correlations: 0.17 (PCV13), −0.18 (PCV10)
correlations: 0.32 (PCV13), −0.24 (PCV10)
Serotype 9V
correlations: 0.26 (PCV13), 0.33 (PCV10)
correlations: −0.02 (PCV13), −0.15 (PCV10)
correlations: −0.01 (PCV13), 0.02 (PCV10)
Serotype 14
correlations: 0.17 (PCV13), 0.61 (PCV10)
correlations: 0.05 (PCV13), −0.27 (PCV10)
correlations: 0.03 (PCV13), −0.35 (PCV10)
Serotype 18C
correlations: 0.66 (PCV13), 0.41 (PCV10)
correlations: 0.40 (PCV13), −0.07 (PCV10)
correlations: 0.32 (PCV13), 0.22 (PCV10)
log(OPA titer)
log(IgG)
log(Avidity index)
log(Avitiy index)

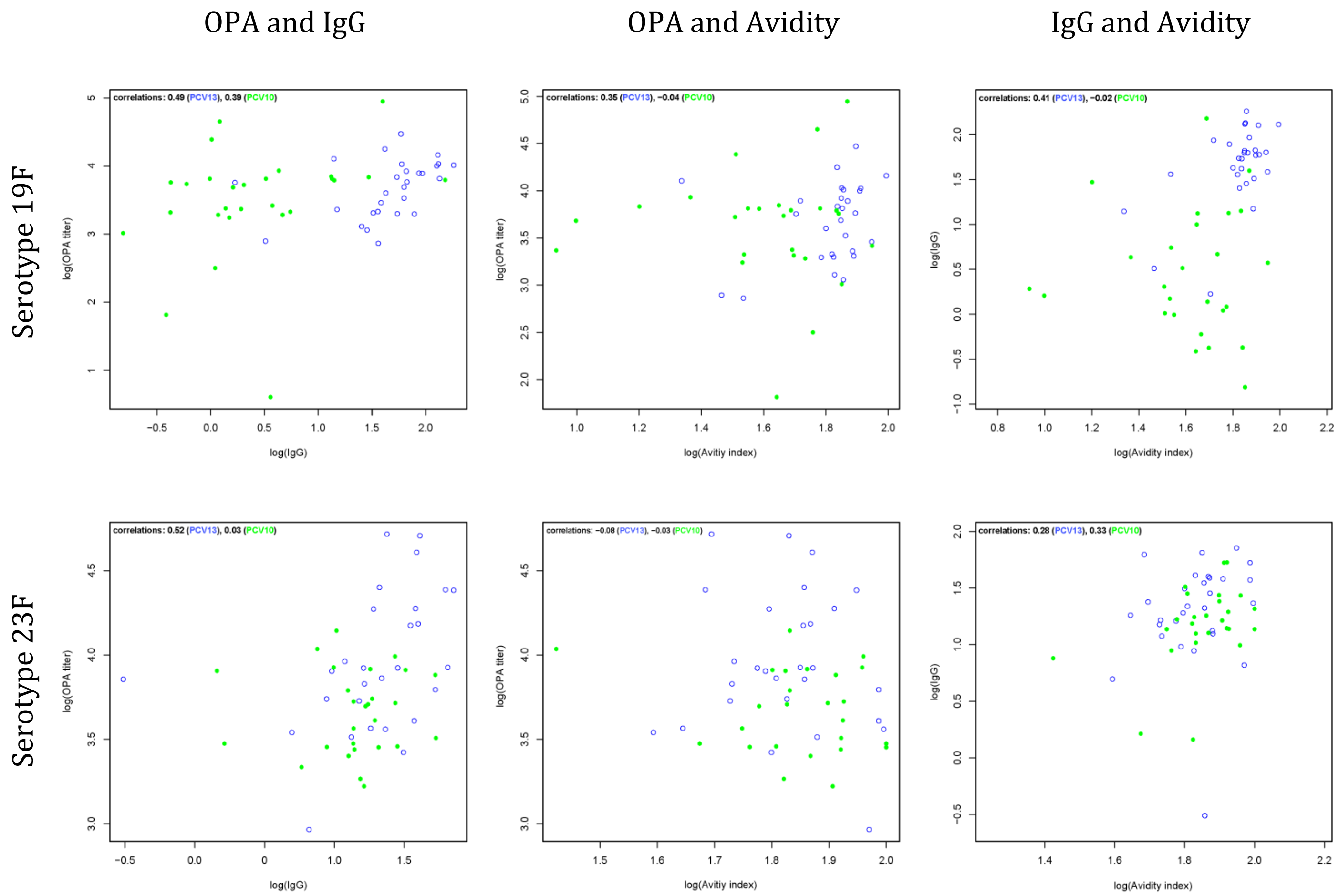

OPA and IgG
OPA and Avidity
IgG and Avidity
Serotype 19F
Serotype 23F
correlations: 0.49 (PCV13), 0.39 (PCV10)
correlations: 0.35 (PCV13), −0.04 (PCV10)
correlations: 0.41 (PCV13), −0.02 (PCV10)
correlations: 0.52 (PCV13), 0.03 (PCV10)
correlations: −0.08 (PCV13), −0.03 (PCV10)
correlations: 0.28 (PCV13), 0.33 (PCV10)
log(OPA titer)
log(IgG)
log(Avitiy index)
log(Avidity index)

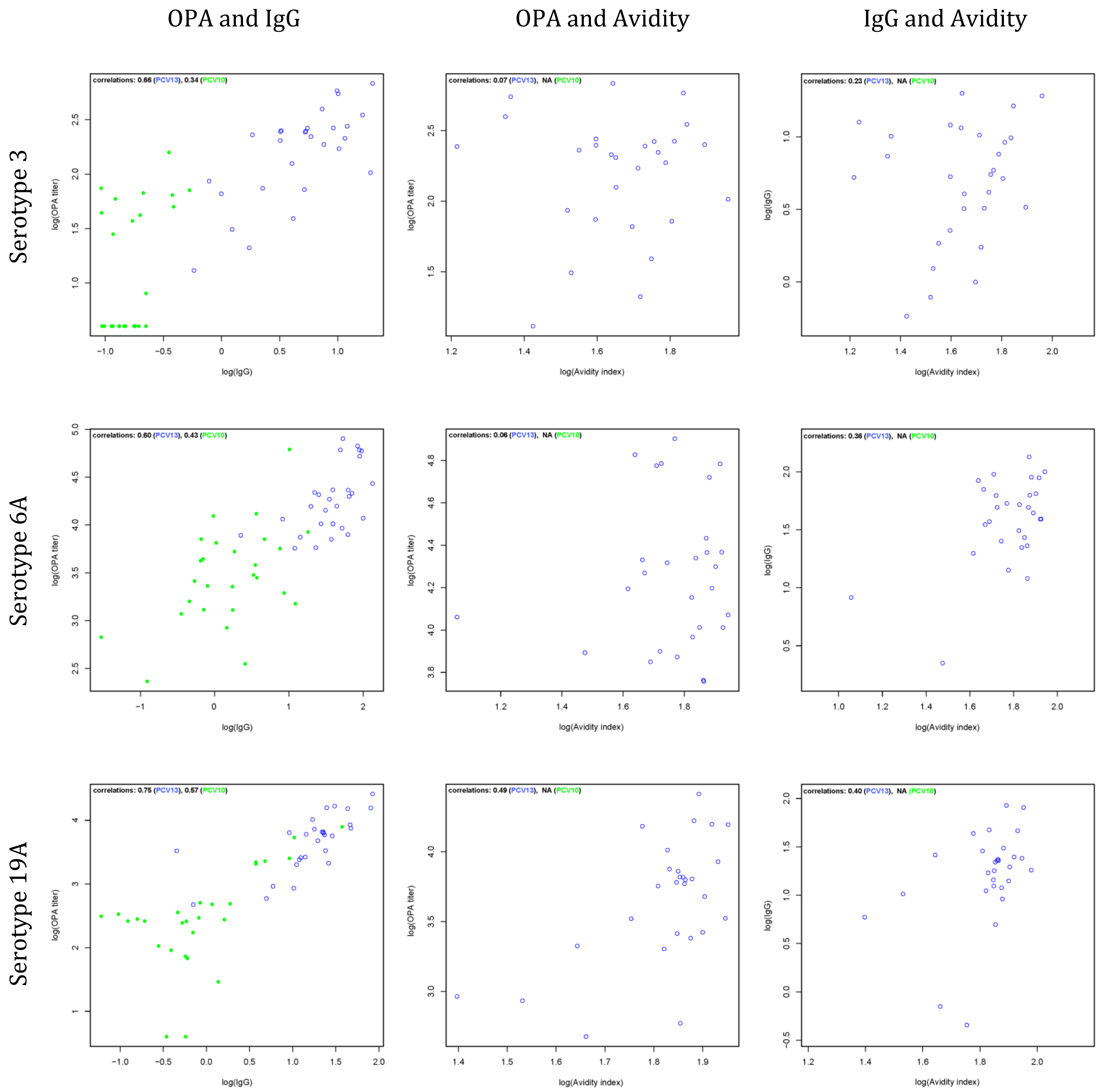

OPA and IgG
OPA and Avidity
IgG and Avidity
Serotype 3
Serotype 6A
Serotype 19A
correlations: 0.66 (PCV13), 0.34 (PCV10)
correlations: 0.07 (PCV13), NA (PCV10)
correlations: 0.23 (PCV13), NA (PCV10)
correlations: 0.60 (PCV13), 0.43 (PCV10)
correlations: 0.06 (PCV13), NA (PCV10)
correlations: 0.36 (PCV13), NA (PCV10)
correlations: 0.75 (PCV13), 0.57 (PCV10)
correlations: 0.49 (PCV13), NA (PCV10)
correlations: 0.40 (PCV13), NA (PCV10)
log(OPA titer)
log(IgG)
log(Avidity index)
